# A digital collection of rare and endangered lemurs and other primates from the Duke Lemur Center

**DOI:** 10.1371/journal.pone.0219411

**Published:** 2019-11-26

**Authors:** Gabriel S. Yapuncich, Addison D. Kemp, Darbi M. Griffith, Justin T. Gladman, Erin Ehmke, Doug M. Boyer

**Affiliations:** 1 Department of Evolutionary Anthropology, Duke University, Durham, North Carolina, United States of America; 2 Department of Anthropology, University of Texas, Austin, Texas, United States of America; 3 Shared Materials Instrumentation Facility (SMIF), Duke University, Durham, North Carolina, United States of America; 4 Duke Lemur Center, Duke University, Durham, North Carolina, United States of America; Monash University, AUSTRALIA

## Abstract

Scientific study of lemurs, a group of primates found only on Madagascar, is crucial for understanding primate evolution. Unfortunately, lemurs are among the most endangered animals in the world, so there is a strong impetus to maximize as much scientific data as possible from available physical specimens. MicroCT scanning efforts at Duke University have resulted in scans of more than 100 strepsirrhine cadavers representing 18 species from the Duke Lemur Center. An error study of the microCT scanner recovered less than 0.3% error at multiple resolution levels. Scans include specimen overviews and focused, high-resolution selections of complex anatomical regions (e.g., cranium, hands, feet). Scans have been uploaded to MorphoSource, an online digital repository for 3D data. As captive (but free ranging) individuals, these specimens have a wealth of associated information that is largely unavailable for wild populations, including detailed life history data. This digital collection maximizes the information obtained from rare and endangered animals with minimal degradation of the original specimens.

## Introduction

Lemurs, a group of primates endemic to Madagascar, are an important group of animals for understanding the evolutionary history and adaptive origins of primates. Unfortunately, they are among the most endangered mammals in the world, with 94% of lemur species threatened by extinction [1]. Continued habitat degradation and fragmentation, illegal poaching, and challenging economic and political circumstances in Madagascar mean that lemurs are likely to remain under acute threat in the foreseeable future [1]. While conservation groups have developed several local, site-specific action plans [2], protecting and studying these animals requires multiple strategies both in Madagascar and internationally.

The Duke Lemur Center (formerly the Duke University Primate Center) is a prime example of an alternative approach to the conservation and scientific study of lemurs. Founded in 1966 in Durham, North Carolina, the Duke Lemur Center (DLC) was established to operate as a “living laboratory” and permit non-invasive study of rare primates, including galagos, lorises, and lemurs (which together comprise the primate suborder Strepsirrhini). Over its history, the DLC has housed more than 4,000 individuals from 39 primate species, and currently houses nearly 240 individuals from 17 strepsirrhine species. The DLC is involved in multiple lemur conservation efforts, including 1) managing several breeding populations as an Association of Zoos and Aquariums accredited institution; 2) the SAVA conservation program, a community-based approach to sustainable forest management and economic improvement in northern Madagascar; and 3) working with the Malagasy government to develop animal husbandry, welfare, and breeding programs for ex-situ lemur populations in Madagascar.

While the cofounders of the DLC, Dr. John Buettner-Janusch and Dr. Peter Klopfer, ran research programs focused on genetics and behaviour respectively, the DLC’s unique resources have provided data for a wide variety of scientific fields, including anatomy and physiology [e.g., 3–6], social ecology [e.g., 7–8], cognition [e.g., 9–10, biomechanics [e.g., 11–12], molecular biology [e.g., 13–14], and palaeontology [e.g., 15–18]. The importance and rarity of the animals housed at the DLC necessitates thorough and effective use in educational and research initiatives, and this spirit of efficiency extends to treatment of deceased individuals. When an animal dies at the DLC (most frequently of old age), veterinary staff perform necropsies to remove internal organs and then preserve cadavers in cold storage for future research purposes. There are currently more than 400 cadavers in storage. However, because DLC cadaveric specimens are available for destructive sampling for research purposes [e.g., 19–21], the total information preserved by each specimen decreases over time. Digitizing the DLC’s cadaveric collection presents an opportunity to preserve hard tissue data without degrading the specimen’s anatomical integrity, thereby increasing the educational and scientific value of these rare and endangered animals.

Here we present an open access 3D digital collection of microCT scan data representing 113 adult individuals from the Duke Lemur Center. The data presented here were generated by GSY and ADK for use in their dissertations [22, 23]. All scans are publicly available on MorphoSource.org [24], an online repository specifically designed to archive 3D data. At the time of manuscript preparation, the collection consists of 483 TIFF volume stacks and 374 surface files generated from the volume data. The collection will continue to grow with future scanning efforts at Duke and potentially from contributions made by other researchers (in the form of new scans or surface files derived from the current collection). Primate cadavers have long been recognized as valuable scientific resources that could be utilized much more efficiently [25, 26], provided scientists motivated by different research questions could coordinate specimen access. The digital collection presented here follows several recent efforts to digitize and publish unique and valuable datasets [27–29].

## Materials and methods

### DLC cadaveric collection

**Fig 1:**
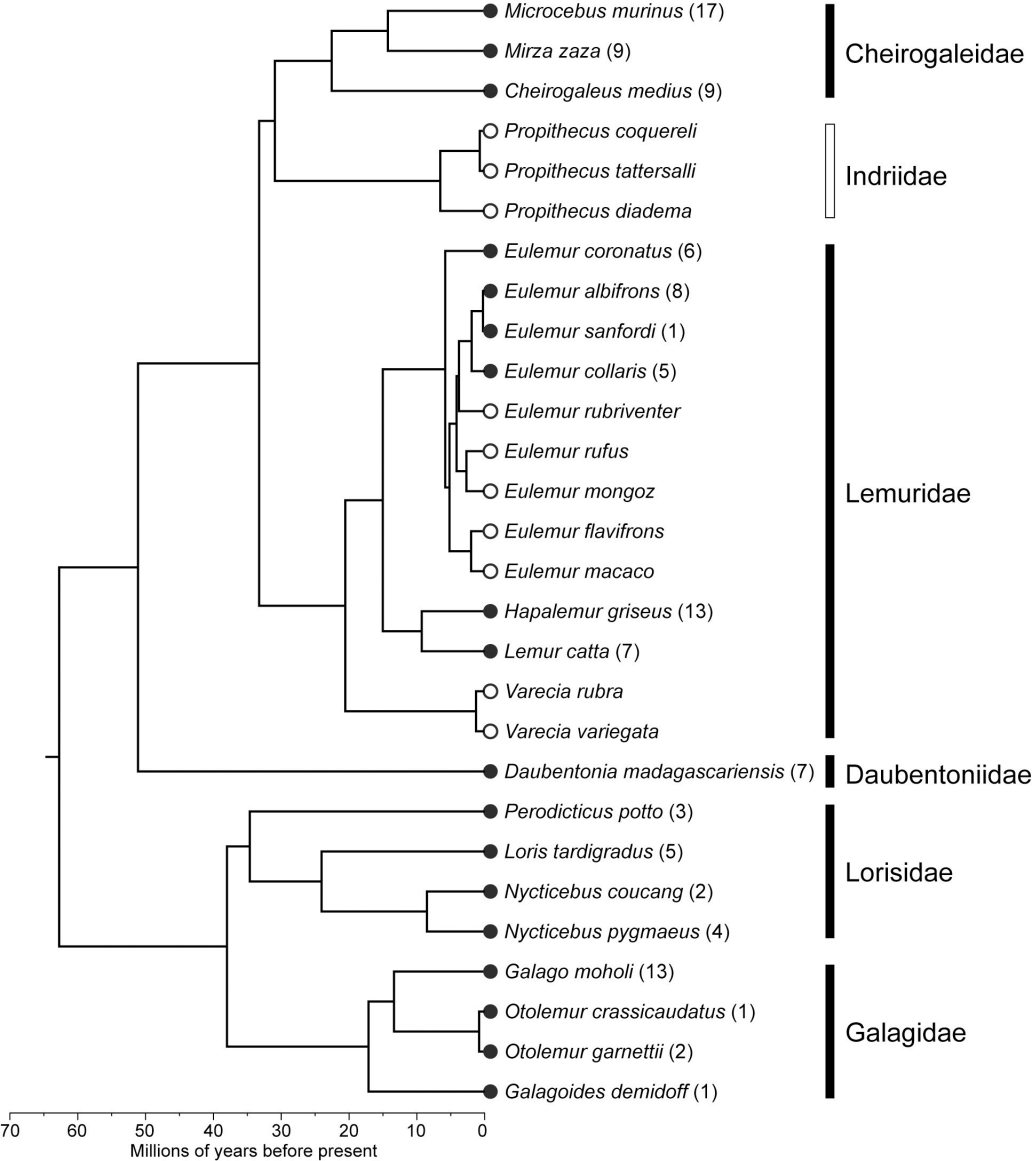
Strepsirrhine phylogeny and number of individuals included in this microCT collection. Dark circles and bars indicate taxa currently represented in the collection. Open circles and bars represent taxa housed at DLC but not currently scanned. Chronogram downloaded from 10ktrees.org, version 3 [39].

A total of 483 microCT (μCT) scans of strepsirrhine primates housed at the Duke Lemur Center were performed at the Shared Materials Instrumentation Facility at Duke University. Specimens represent both major clades of strepsirrhine primates, including 82 lemurs, 17 galagos, and 14 lorises (Fig 1). Among these individuals, two lemurs and five loris specimens were iodine-stained to permit visualization of soft tissue anatomy. Currently, the sample is biased toward individuals less than 3 kg. Individuals of larger species housed at the DLC (e.g., *Propithecus* and *Varecia*) have not yet been μCT scanned, although methods developed in the course of this project will allow them to be scanned at high resolutions in the near future. Additional demographic information for most specimens is available in Zehr et al. [30]. Table 1 provides a summary of the sample by species, and individual specimens, scanning parameters and digital object identifiers (DOIs) for all currently available TIFF volumes are provided in Table 2.

**Table 1:** Summary of sample by species.

Species	*n* individuals	*k* scans	Iodine specimens
*Cheirogaleus medius*	9	35	DLC 1657m
*Daubentonia madagascariensis*	7	35	
*Eulemur coronatus*	6	23	
*Eulemur albifrons*	8	31	
*Eulemur collaris*	5	18	
*Eulemur sanfordi*	1	5	
*Galago moholi*	13	53	
*Galagoides demidovii*	1	4	
*Hapalemur griseus*	13	57	
*Lemur catta*	7	35	
*Loris tardigradus*	5	23	DLC 977f; 1902f
*Microcebus murinus*	17	72	
*Mirza zaza*	9	38	DLC 373f
*Nycticebus coucang*	2	10	DLC 993m
*Nycticebus pygmaeus*	4	15	
*Otolemur crassicaudatus*	1	6	DLC 1715f
*Otolemur garnetii*	2	10	
*Perodicticus potto*	3	13	DLC 917m
Total	113	483	7

**Table 2:** Specimens, scan names, scan parameters, and DOIs for all TIFF stacks included in this collection. Abbreviations: mm, millimeters; kV, kilovolts; uA, microamps; GB, gigabytes; MB, megabytes.

Specimen	*Species*	Title	DOI	File size	X resolution (mm)	Y resolution (mm)	Z resolution (mm)	Voltage (kV)	Amperage (μA)	Watts (W)	Projections	Copyright
DLC 1607m	*Cheirogaleus medius*	Foot Zipped TIFF Stack	https://doi.org/10.17602/M2/M14367	1.06 GB	0.025212629	0.025212629	0.025212629	140	115	16.1	2000	Duke Lemur Center CC BY-NC
DLC 1607m	*Cheirogaleus medius*	Hand Zipped TIFF Stack	https://doi.org/10.17602/M2/M14370	799.38 MB	0.026286393	0.026286393	0.026286393	140	115	16.1	2000	Duke Lemur Center CC BY-NC
DLC 1607m	*Cheirogaleus medius*	Skull Zipped TIFF Stack	https://doi.org/10.17602/M2/M14380	2.75 GB	0.029462179	0.029462179	0.029462179	140	115	16.1	2000	Duke Lemur Center CC BY-NC
DLC 1607m	*Cheirogaleus medius*	Full Body Zipped TIFF Stack	https://doi.org/10.17602/M2/M16237	5.85 GB	0.04493203	0.04493203	0.04493203	156	112	17.47	2000	Duke Lemur Center CC BY-NC
DLC 1636m	*Cheirogaleus medius*	Hand Zipped TIFF Stack	https://doi.org/10.17602/M2/M14382	872.5 MB	0.024448479	0.024448479	0.024448479	150	100	15	2000	Duke Lemur Center CC BY-NC
DLC 1636m	*Cheirogaleus medius*	Foot Zipped TIFF Stack	https://doi.org/10.17602/M2/M14383	937.26 MB	0.032077279	0.032077279	0.032077279	150	100	15	2000	Duke Lemur Center CC BY-NC
DLC 1636m	*Cheirogaleus medius*	Skull Zipped TIFF Stack	https://doi.org/10.17602/M2/M14389	2.46 GB	0.030689647	0.030689647	0.030689647	150	100	15	2000	Duke Lemur Center CC BY-NC
DLC 1636m	*Cheirogaleus medius*	Full Body Zipped TIFF Stack	https://doi.org/10.17602/M2/M16146	7.17 GB	0.049688201	0.049688201	0.049688201	140	115	16.1	2000	Duke Lemur Center CC BY-NC
DLC 1653f	*Cheirogaleus medius*	Hand Zipped TIFF Stack	https://doi.org/10.17602/M2/M14388	1.44 GB	0.018531365	0.018531365	0.018531365	155	120	18.6	2000	Duke Lemur Center CC BY-NC
DLC 1653f	*Cheirogaleus medius*	Skull Zipped TIFF Stack	https://doi.org/10.17602/M2/M16231	4.33 GB	0.027835561	0.027835561	0.027835561	155	120	18.6	2000	Duke Lemur Center CC BY-NC
DLC 1653f	*Cheirogaleus medius*	Full Body Zipped TIFF Stack	https://doi.org/10.17602/M2/M26259	949.68 MB	0.055180769	0.055180769	0.055180769	155	120	18.6	2000	Duke Lemur Center CC BY-NC
DLC 1657m	*Cheirogaleus medius*	Full Body Zipped TIFF Stack	https://doi.org/10.17602/M2/M39697	2.85 GB	0.051564646	0.051564646	0.051564646	145	157	22.77	2000	Duke Lemur Center CC BY-NC
DLC 1657m	*Cheirogaleus medius*	Foot Zipped TIFF Stack	https://doi.org/10.17602/M2/M39699	820.52 MB	0.022253715	0.022253715	0.022253715	140	169	23.66	2000	Duke Lemur Center CC BY-NC
DLC 1657m	*Cheirogaleus medius*	Forelimb Zipped TIFF Stack	https://doi.org/10.17602/M2/M39702	1.53 GB	0.033372468	0.033372468	0.033372468	148	231	34.19	2000	Duke Lemur Center CC BY-NC
DLC 1657m	*Cheirogaleus medius*	Hand Zipped TIFF Stack	https://doi.org/10.17602/M2/M39705	788.6 MB	0.016755471	0.016755471	0.016755471	203	132	26.8	2000	Duke Lemur Center CC BY-NC
DLC 1657m	*Cheirogaleus medius*	Hindlimb Zipped TIFF Stack	https://doi.org/10.17602/M2/M39707	1.63 GB	0.02942261	0.02942261	0.02942261	145	126	18.27	2000	Duke Lemur Center CC BY-NC
DLC 1657m	*Cheirogaleus medius*	Skull Zipped TIFF Stack	https://doi.org/10.17602/M2/M39712	1.74 GB	0.026287325	0.026287325	0.026287325	163	241	39.28	2000	Duke Lemur Center CC BY-NC
DLC 3640f	*Cheirogaleus medius*	Hand Zipped TIFF Stack	https://doi.org/10.17602/M2/M15238	2.92 GB	0.017541829	0.017541829	0.017541829	150	111	16.65	2000	Duke Lemur Center CC BY-NC
DLC 3640f	*Cheirogaleus medius*	Foot Zipped TIFF Stack	https://doi.org/10.17602/M2/M15240	3.27 GB	0.01787997	0.01787997	0.01787997	150	111	16.65	2000	Duke Lemur Center CC BY-NC
DLC 3640f	*Cheirogaleus medius*	Skull Zipped TIFF Stack	https://doi.org/10.17602/M2/M15246	3.69 GB	0.023770703	0.023770703	0.023770703	150	146	21.9	2000	Duke Lemur Center CC BY-NC
DLC 3640f	*Cheirogaleus medius*	Full Body Zipped TIFF Stack	https://doi.org/10.17602/M2/M16967	944.31 MB	0.048064884	0.048064884	0.048064884	150	146	21.9	1800	Duke Lemur Center CC BY-NC
DLC 613f	*Cheirogaleus medius*	Foot Zipped TIFF Stack	https://doi.org/10.17602/M2/M15349	2.52 GB	0.01618115	0.01618115	0.01618115	155	120	18.6	2000	Duke Lemur Center CC BY-NC
DLC 613f	*Cheirogaleus medius*	Hand Zipped TIFF Stack	https://doi.org/10.17602/M2/M16117	2.23 GB	0.013749437	0.013749437	0.013749437	155	120	18.6	2000	Duke Lemur Center CC BY-NC
DLC 613f	*Cheirogaleus medius*	Full Body Zipped TIFF Stack	https://doi.org/10.17602/M2/M16893	1.12 GB	0.046091117	0.046091117	0.046091117	155	120	18.6	2000	Duke Lemur Center CC BY-NC
DLC 656m	*Cheirogaleus medius*	Hand Zipped TIFF Stack	https://doi.org/10.17602/M2/M15350	2.36 GB	0.016657572	0.016657572	0.016657572	155	120	18.6	2000	Duke Lemur Center CC BY-NC
DLC 656m	*Cheirogaleus medius*	Foot Zipped TIFF Stack	https://doi.org/10.17602/M2/M15354	2.68 GB	0.018231485	0.018231485	0.018231485	155	120	18.6	2000	Duke Lemur Center CC BY-NC
DLC 656m	*Cheirogaleus medius*	Full Body Zipped TIFF Stack	https://doi.org/10.17602/M2/M17006	1.16 GB	0.049809046	0.049809046	0.049809046	155	120	18.6	2000	Duke Lemur Center CC BY-NC
DLC 684f	*Cheirogaleus medius*	Foot Zipped TIFF Stack	https://doi.org/10.17602/M2/M15364	1.32 GB	0.023747068	0.023747068	0.023747068	140	115	16.1	2000	Duke Lemur Center CC BY-NC
DLC 684f	*Cheirogaleus medius*	Hand Zipped TIFF Stack	https://doi.org/10.17602/M2/M15365	1.6 GB	0.022694951	0.022694951	0.022694951	140	115	16.1	2000	Duke Lemur Center CC BY-NC
DLC 684f	*Cheirogaleus medius*	Skull Zipped TIFF Stack	https://doi.org/10.17602/M2/M15366	2.01 GB	0.032473497	0.032473497	0.032473497	140	115	16.1	2000	Duke Lemur Center CC BY-NC
DLC 684f	*Cheirogaleus medius*	Full Body Zipped TIFF Stack	https://doi.org/10.17602/M2/M31312	6.74 GB	0.048452061	0.048452061	0.048452061	140	115	16.1	2000	Duke Lemur Center CC BY-NC
DLC 687f	*Cheirogaleus medius*	Foot Zipped TIFF Stack	https://doi.org/10.17602/M2/M15385	3.08 GB	0.018175488	0.018175488	0.018175488	155	120	18.6	2000	Duke Lemur Center CC BY-NC
DLC 687f	*Cheirogaleus medius*	Hands Zipped TIFF Stack	https://doi.org/10.17602/M2/M15386	2.93 GB	0.01998697	0.01998697	0.01998697	155	120	18.6	2000	Duke Lemur Center CC BY-NC
DLC 687f	*Cheirogaleus medius*	Skull Zipped TIFF Stack	https://doi.org/10.17602/M2/M15397	4.25 GB	0.029686933	0.029686933	0.029686933	155	120	18.6	2000	Duke Lemur Center CC BY-NC
DLC 687f	*Cheirogaleus medius*	Full Body Zipped TIFF Stack	https://doi.org/10.17602/M2/M34267	8.91 GB	0.043963458	0.043963458	0.043963458	155	120	18.6	2000	Duke Lemur Center CC BY-NC
DLC 6454f	*Daubentonia madagascariensis*	Basicranium Zipped TIFF Stack	https://doi.org/10.17602/M2/M31763	4.85 GB	0.036600497	0.036600497	0.036600497	105	338	35.49	2000	Duke Lemur Center CC BY-NC
DLC 6454f	*Daubentonia madagascariensis*	Cranium Zipped TIFF Stack	https://doi.org/10.17602/M2/M32046	3.22 GB	0.055889439	0.055889439	0.055889439	80	528	42.24	2000	Duke Lemur Center CC BY-NC
DLC 6454f	*Daubentonia madagascariensis*	Feet Zipped TIFF Stack	https://doi.org/10.17602/M2/M32049	2.48 GB	0.056194843	0.056194843	0.056194843	80	528	42.24	2000	Duke Lemur Center CC BY-NC
DLC 6454f	*Daubentonia madagascariensis*	Hands Zipped TIFF Stack	https://doi.org/10.17602/M2/M32053	2.63 GB	0.050790539	0.050790539	0.050790539	80	528	42.24	2000	Duke Lemur Center CC BY-NC
DLC 6454f	*Daubentonia madagascariensis*	Full Body Zipped TIFF Stack	https://doi.org/10.17602/M2/M32055	5.95 GB	0.097271818	0.097271818	0.097271818	95	495	47.03	1800	Duke Lemur Center CC BY-NC
DLC 6604m	*Daubentonia madagascariensis*	Full Body Zipped TIFF Stack	https://doi.org/10.17602/M2/M24727	6.12 GB	0.105899701	0.105899701	0.105899701	120	396	47.52	2000	Duke Lemur Center CC BY-NC
DLC 6604m	*Daubentonia madagascariensis*	Feet Zipped TIFF Stack	https://doi.org/10.17602/M2/M25460	1.69 GB	0.068388529	0.068388529	0.068388529	100	430	43	2000	Duke Lemur Center CC BY-NC
DLC 6604m	*Daubentonia madagascariensis*	Skull Zipped TIFF Stack	https://doi.org/10.17602/M2/M25461	2.06 GB	0.067548797	0.067548797	0.067548797	110	401	44.11	2000	Duke Lemur Center CC BY-NC
DLC 6604m	*Daubentonia madagascariensis*	Hands Zipped TIFF Stack	https://doi.org/10.17602/M2/M25464	3.41 GB	0.06734892	0.06734892	0.06734892	110	401	44.11	2000	Duke Lemur Center CC BY-NC
DLC 6725m	*Daubentonia madagascariensis*	Cranium Zipped TIFF stack	https://doi.org/10.17602/M2/M73679	942.39 MB	0.053337122	0.053337122	0.053337122	105	377	39.59	2400	Duke Lemur Center CC BY-NC
DLC 6725m	*Daubentonia madagascariensis*	Feet Zipped TIFF stack	https://doi.org/10.17602/M2/M73689	269.46 MB	0.058621731	0.058621731	0.058621731	105	246	25.83	2000	Duke Lemur Center CC BY-NC
DLC 6725m	*Daubentonia madagascariensis*	Hands Zipped TIFF stack	https://doi.org/10.17602/M2/M73697	634.99 MB	0.067034011	0.067034011	0.067034011	105	377	39.59	2000	Duke Lemur Center CC BY-NC
DLC 6788m	*Daubentonia madagascariensis*	Feet Zipped TIFF Stack	https://doi.org/10.17602/M2/M14502	2.49 GB	0.071475327	0.071475327	0.071475327	140	262	36.68	2000	Duke Lemur Center CC BY-NC
DLC 6788m	*Daubentonia madagascariensis*	Hands Zipped TIFF Stack	https://doi.org/10.17602/M2/M14513	3.31 GB	0.057212051	0.057212051	0.057212051	140	262	36.68	2000	Duke Lemur Center CC BY-NC
DLC 6788m	*Daubentonia madagascariensis*	Leg Zipped TIFF Stack	https://doi.org/10.17602/M2/M14514	3.72 GB	0.103460729	0.103460729	0.103460729	130	308	40.04	2000	Duke Lemur Center CC BY-NC
DLC 6788m	*Daubentonia madagascariensis*	Lower Zipped TIFF Stack	https://doi.org/10.17602/M2/M14535	4 GB	0.103460729	0.103460729	0.103460729	130	308	40.04	2000	Duke Lemur Center CC BY-NC
DLC 6788m	*Daubentonia madagascariensis*	Skull Zipped TIFF Stack	https://doi.org/10.17602/M2/M14545	2.66 GB	0.072993837	0.072993837	0.072993837	140	262	36.68	2000	Duke Lemur Center CC BY-NC
DLC 6788m	*Daubentonia madagascariensis*	Upper Zipped TIFF Stack	https://doi.org/10.17602/M2/M14546	4.18 GB	0.103460729	0.103460729	0.103460729	130	308	40.04	2000	Duke Lemur Center CC BY-NC
DLC 6866f	*Daubentonia madagascariensis*	Full Body Zipped TIFF Stack	https://doi.org/10.17602/M2/M25337	6.28 GB	0.105899692	0.105899692	0.105899692	120	396	47.52	2000	Duke Lemur Center CC BY-NC
DLC 6866f	*Daubentonia madagascariensis*	Foot Zipped TIFF Stack	https://doi.org/10.17602/M2/M25452	859.13 MB	0.075191759	0.075191759	0.075191759	100	417	41.7	2000	Duke Lemur Center CC BY-NC
DLC 6866f	*Daubentonia madagascariensis*	Foot Zipped TIFF Stack	https://doi.org/10.17602/M2/M25453	823.18 MB	0.075191759	0.075191759	0.075191759	100	417	41.7	2000	Duke Lemur Center CC BY-NC
DLC 6866f	*Daubentonia madagascariensis*	Hands Zipped TIFF Stack	https://doi.org/10.17602/M2/M25456	2.99 GB	0.068061851	0.068061851	0.068061851	100	417	41.7	2000	Duke Lemur Center CC BY-NC
DLC 6866f	*Daubentonia madagascariensis*	Skull Zipped TIFF Stack	https://doi.org/10.17602/M2/M25457	2.33 GB	0.061293066	0.061293066	0.061293066	100	417	41.7	2000	Duke Lemur Center CC BY-NC
DLC 6915m	*Daubentonia madagascariensis*	Hand Zipped TIFF Stack	https://doi.org/10.17602/M2/M14562	1.56 GB	0.053757131	0.053757131	0.053757131	115	354	40.71	1800	Duke Lemur Center CC BY-NC
DLC 6915m	*Daubentonia madagascariensis*	Hand/Foot Zipped TIFF Stack	https://doi.org/10.17602/M2/M14564	2.38 GB	0.05461977	0.05461977	0.05461977	115	354	40.71	1800	Duke Lemur Center CC BY-NC
DLC 6915m	*Daubentonia madagascariensis*	Lower Zipped TIFF Stack	https://doi.org/10.17602/M2/M14565	2.24 GB	0.110741361	0.110741361	0.110741361	115	354	40.71	1800	Duke Lemur Center CC BY-NC
DLC 6915m	*Daubentonia madagascariensis*	Tail Zipped TIFF Stack	https://doi.org/10.17602/M2/M14571	1.68 GB	0.110741361	0.110741361	0.110741361	115	354	40.71	1800	Duke Lemur Center CC BY-NC
DLC 6915m	*Daubentonia madagascariensis*	Skull Zipped TIFF Stack	https://doi.org/10.17602/M2/M14577	3.01 GB	0.051406145	0.051406145	0.051406145	115	354	40.71	2000	Duke Lemur Center CC BY-NC
DLC 6915m	*Daubentonia madagascariensis*	Tail Zipped TIFF Stack	https://doi.org/10.17602/M2/M14578	931.79 MB	0.110741361	0.110741361	0.110741361	115	354	40.71	1800	Duke Lemur Center CC BY-NC
DLC 6915m	*Daubentonia madagascariensis*	Upper Zipped TIFF Stack	https://doi.org/10.17602/M2/M14587	1.89 GB	0.110741361	0.110741361	0.110741361	115	354	40.71	1800	Duke Lemur Center CC BY-NC
DLC 6915m	*Daubentonia madagascariensis*	Foot Zipped TIFF Stack	https://doi.org/10.17602/M2/M33905	1.58 GB	0.053148061	0.053148061	0.053148061	115	354	40.71	1800	Duke Lemur Center CC BY-NC
DLC 6941m	*Daubentonia madagascariensis*	Hands Zipped TIFF Stack	https://doi.org/10.17602/M2/M24649	2.03 GB	0.064287297	0.064287297	0.064287297	100	417	41.7	1800	Duke Lemur Center CC BY-NC
DLC 6941m	*Daubentonia madagascariensis*	Feet Zipped TIFF Stack	https://doi.org/10.17602/M2/M24657	2.97 GB	0.075697608	0.075697608	0.075697608	100	417	41.7	1800	Duke Lemur Center CC BY-NC
DLC 6941m	*Daubentonia madagascariensis*	Skull Zipped TIFF Stack	https://doi.org/10.17602/M2/M24658	1.72 GB	0.061931368	0.061931368	0.061931368	100	417	41.7	2000	Duke Lemur Center CC BY-NC
DLC 6941m	*Daubentonia madagascariensis*	Full Body Zipped TIFF Stack	https://doi.org/10.17602/M2/M24659	5.34 GB	0.107117295	0.107117295	0.107117295	110	418	45.98	1800	Duke Lemur Center CC BY-NC
DLC 5937f	*Eulemur coronatus*	Leg and feet Zipped TIFF Stack	https://doi.org/10.17602/M2/M26983	689.47 MB	0.076826893	0.076826893	0.076826893	90	393	35.37	2000	Duke Lemur Center CC BY-NC
DLC 5937f	*Eulemur coronatus*	Full Body Zipped TIFF Stack	https://doi.org/10.17602/M2/M33678	2.79 GB	0.107995905	0.107995905	0.107995905	95	568	53.96	1800	Duke Lemur Center CC BY-NC
DLC 5937f	*Eulemur coronatus*	Hands Zipped TIFF Stack	https://doi.org/10.17602/M2/M33680	649.9 MB	0.107995905	0.107995905	0.107995905	95	568	53.96	1800	Duke Lemur Center CC BY-NC
DLC 5937f	*Eulemur coronatus*	Skull Zipped TIFF Stack	https://doi.org/10.17602/M2/M33682	1.9 GB	0.107995905	0.107995905	0.107995905	95	568	53.96	1800	Duke Lemur Center CC BY-NC
DLC 5937f	*Eulemur coronatus*	Feet Zipped TIFF Stack	https://doi.org/10.17602/M2/M33684	553.02 MB	0.076826893	0.076826893	0.076826893	90	393	35.37	2000	Duke Lemur Center CC BY-NC
DLC 6034m	*Eulemur coronatus*	Full Body Zipped TIFF Stack	https://doi.org/10.17602/M2/M27505	1.95 GB	0.093560301	0.093560301	0.093560301	100	435	43.5	1800	Duke Lemur Center CC BY-NC
DLC 6034m	*Eulemur coronatus*	Feet Zipped TIFF Stack	https://doi.org/10.17602/M2/M29671	966.42 MB	0.053041793	0.053041793	0.053041793	90	416	37.44	1800	Duke Lemur Center CC BY-NC
DLC 6034m	*Eulemur coronatus*	Hands Zipped TIFF Stack	https://doi.org/10.17602/M2/M29673	362.79 MB	0.05192272	0.05192272	0.05192272	90	416	37.44	1800	Duke Lemur Center CC BY-NC
DLC 6034m	*Eulemur coronatus*	Skull Zipped TIFF Stack	https://doi.org/10.17602/M2/M29677	1.67 GB	0.046532523	0.046532523	0.046532523	90	416	37.44	1800	Duke Lemur Center CC BY-NC
DLC 6035m	*Eulemur coronatus*	Feet Zipped TIFF Stack	https://doi.org/10.17602/M2/M29679	642.63 MB	0.052615654	0.052615654	0.052615654	90	416	37.44	1800	Duke Lemur Center CC BY-NC
DLC 6035m	*Eulemur coronatus*	Hand Zipped TIFF Stack	https://doi.org/10.17602/M2/M29681	820.72 MB	0.05261565	0.05261565	0.05261565	90	416	37.44	1800	Duke Lemur Center CC BY-NC
DLC 6035m	*Eulemur coronatus*	Full Body Zipped TIFF Stack	https://doi.org/10.17602/M2/M29685	2.45 GB	0.110741448	0.110741448	0.110741448	100	435	43.5	1800	Duke Lemur Center CC BY-NC
DLC 6035m	*Eulemur coronatus*	Skull Zipped TIFF Stack	https://doi.org/10.17602/M2/M29688	1.56 GB	0.04962183	0.04962183	0.04962183	85	432	36.72	1900	Duke Lemur Center CC BY-NC
DLC 6177f	*Eulemur coronatus*	Full Body Zipped TIFF Stack	https://doi.org/10.17602/M2/M31062	2.02 GB	0.102041118	0.102041118	0.102041118	105	439	46.1	1800	Duke Lemur Center CC BY-NC
DLC 6177f	*Eulemur coronatus*	Hands Zipped TIFF Stack	https://doi.org/10.17602/M2/M31064	1.23 GB	0.053221423	0.053221423	0.053221423	90	416	37.44	1800	Duke Lemur Center CC BY-NC
DLC 6177f	*Eulemur coronatus*	Feet Zipped TIFF Stack	https://doi.org/10.17602/M2/M31067	1.34 GB	0.053221423	0.053221423	0.053221423	90	416	37.44	1800	Duke Lemur Center CC BY-NC
DLC 6177f	*Eulemur coronatus*	Skull Zipped TIFF Stack	https://doi.org/10.17602/M2/M31071	1.62 GB	0.053221423	0.053221423	0.053221423	90	416	37.44	1800	Duke Lemur Center CC BY-NC
DLC 6366f	*Eulemur coronatus*	Full Body Zipped TIFF Stack	https://doi.org/10.17602/M2/M31745	2.64 GB	0.106218575	0.106218575	0.106218575	100	470	47	1800	Duke Lemur Center CC BY-NC
DLC 6366f	*Eulemur coronatus*	Skull Zipped TIFF Stack	https://doi.org/10.17602/M2/M31746	1.05 GB	0.065021768	0.065021768	0.065021768	90	381	34.29	1800	Duke Lemur Center CC BY-NC
DLC 6366f	*Eulemur coronatus*	Hand Zipped TIFF Stack	https://doi.org/10.17602/M2/M31749	432.03 MB	0.056339454	0.056339454	0.056339454	90	381	34.29	1800	Duke Lemur Center CC BY-NC
DLC 6366f	*Eulemur coronatus*	Feet Zipped TIFF Stack	https://doi.org/10.17602/M2/M31753	736.72 MB	0.068660222	0.068660222	0.068660222	90	381	34.29	1800	Duke Lemur Center CC BY-NC
DLC 6441f	*Eulemur coronatus*	Full Body Zipped TIFF Stack	https://doi.org/10.17602/M2/M33961	2.1 GB	0.106753863	0.106753863	0.106753863	95	564	53.58	1800	Duke Lemur Center CC BY-NC
DLC 6441f	*Eulemur coronatus*	Hand Zipped TIFF Stack	https://doi.org/10.17602/M2/M34269	545.51 MB	0.053702723	0.053702723	0.053702723	75	593	44.48	2000	Duke Lemur Center CC BY-NC
DLC 5512f	*Eulemur albifrons*	Feet Zipped TIFF Stack	https://doi.org/10.17602/M2/M29994	1.18 GB	0.061939657	0.061939657	0.061939657	100	315	31.5	2000	Duke Lemur Center CC BY-NC
DLC 5512f	*Eulemur albifrons*	Skull Zipped TIFF Stack	https://doi.org/10.17602/M2/M29996	1.55 GB	0.061939657	0.061939657	0.061939657	100	315	31.5	2000	Duke Lemur Center CC BY-NC
DLC 5512f	*Eulemur albifrons*	Hand Zipped TIFF Stack	https://doi.org/10.17602/M2/M29998	319.91 MB	0.061939664	0.061939664	0.061939664	100	315	31.5	2000	Duke Lemur Center CC BY-NC
DLC 5530m	*Eulemur albifrons*	Full Body Zipped TIFF Stack	https://doi.org/10.17602/M2/M19828	2.74 GB	0.110739912	0.110739912	0.110739912	130	315	40.95	2000	Duke Lemur Center CC BY-NC
DLC 5530m	*Eulemur albifrons*	Hand Zipped TIFF Stack	https://doi.org/10.17602/M2/M19830	373.48 MB	0.055663954	0.055663954	0.055663954	100	370	37	2000	Duke Lemur Center CC BY-NC
DLC 5530m	*Eulemur albifrons*	Skull Zipped TIFF Stack	https://doi.org/10.17602/M2/M19833	2.04 GB	0.055663954	0.055663954	0.055663954	100	370	37	2000	Duke Lemur Center CC BY-NC
DLC 5547f	*Eulemur albifrons*	Full Body Zipped TIFF Stack	https://doi.org/10.17602/M2/M22214	4.99 GB	0.101334296	0.101334296	0.101334296	120	389	46.68	2000	Duke Lemur Center CC BY-NC
DLC 5547f	*Eulemur albifrons*	Skull Zipped TIFF Stack	https://doi.org/10.17602/M2/M22221	1.41 GB	0.069590054	0.069590054	0.069590054	95	410	38.95	2000	Duke Lemur Center CC BY-NC
DLC 5547f	*Eulemur albifrons*	Hands/Feet Zipped TIFF Stack	https://doi.org/10.17602/M2/M22227	1.44 GB	0.065668039	0.065668039	0.065668039	95	410	38.95	2000	Duke Lemur Center CC BY-NC
DLC 576m	*Eulemur albifrons*	Full Body Zipped TIFF Stack	https://doi.org/10.17602/M2/M20366	3.46 GB	0.110739909	0.110739909	0.110739909	130	315	40.95	2000	Duke Lemur Center CC BY-NC
DLC 576m	*Eulemur albifrons*	Skull Zipped TIFF Stack	https://doi.org/10.17602/M2/M20370	1.55 GB	0.051715311	0.051715311	0.051715311	100	370	37	2000	Duke Lemur Center CC BY-NC
DLC 576m	*Eulemur albifrons*	Hand Zipped TIFF Stack	https://doi.org/10.17602/M2/M20372	394.4 MB	0.064558029	0.064558029	0.064558029	100	370	37	2000	Duke Lemur Center CC BY-NC
DLC 576m	*Eulemur albifrons*	Foot Zipped TIFF Stack	https://doi.org/10.17602/M2/M20374	640.48 MB	0.064558029	0.064558029	0.064558029	100	370	37	2000	Duke Lemur Center CC BY-NC
DLC 576m	*Eulemur albifrons*	Foot Zipped TIFF Stack	https://doi.org/10.17602/M2/M20376	813.53 MB	0.064558029	0.064558029	0.064558029	100	370	37	2000	Duke Lemur Center CC BY-NC
DLC 6081m	*Eulemur albifrons*	Full Body Zipped TIFF Stack	https://doi.org/10.17602/M2/M21897	3.61 GB	0.095492706	0.095492706	0.095492706	120	389	46.68	2000	Duke Lemur Center CC BY-NC
DLC 6081m	*Eulemur albifrons*	Skull Zipped TIFF Stack	https://doi.org/10.17602/M2/M22010	2.73 GB	0.052984148	0.052984148	0.052984148	95	410	38.95	2000	Duke Lemur Center CC BY-NC
DLC 6081m	*Eulemur albifrons*	Hand Zipped TIFF Stack	https://doi.org/10.17602/M2/M22016	476.34 MB	0.062752746	0.062752746	0.062752746	95	410	38.95	2000	Duke Lemur Center CC BY-NC
DLC 6081m	*Eulemur albifrons*	Hand Zipped TIFF Stack	https://doi.org/10.17602/M2/M22018	254.66 MB	0.062752746	0.062752746	0.062752746	95	410	38.95	2000	Duke Lemur Center CC BY-NC
DLC 6081m	*Eulemur albifrons*	Feet Zipped TIFF Stack	https://doi.org/10.17602/M2/M22043	1.39 GB	0.062752746	0.062752746	0.062752746	95	410	38.95	2000	Duke Lemur Center CC BY-NC
DLC 6184m	*Eulemur albifrons*	Full Body Zipped TIFF Stack	https://doi.org/10.17602/M2/M24027	4.35 GB	0.10728737	0.10728737	0.10728737	105	487	51.14	1800	Duke Lemur Center CC BY-NC
DLC 6184m	*Eulemur albifrons*	Skull Zipped TIFF Stack	https://doi.org/10.17602/M2/M24158	1.01 GB	0.075670756	0.075670756	0.075670756	100	459	45.9	2000	Duke Lemur Center CC BY-NC
DLC 6184m	*Eulemur albifrons*	Hand Zipped TIFF Stack	https://doi.org/10.17602/M2/M24161	1.01 GB	0.059554111	0.059554111	0.059554111	100	459	45.9	2000	Duke Lemur Center CC BY-NC
DLC 6184m	*Eulemur albifrons*	Hand Zipped TIFF Stack	https://doi.org/10.17602/M2/M30000	329.96 MB	0.065921985	0.065921985	0.065921985	100	459	45.9	2000	Duke Lemur Center CC BY-NC
DLC 6257f	*Eulemur albifrons*	Full Body Zipped TIFF Stack	https://doi.org/10.17602/M2/M22341	4.53 GB	0.108921006	0.108921006	0.108921006	105	462	48.51	1800	Duke Lemur Center CC BY-NC
DLC 6257f	*Eulemur albifrons*	Skull Zipped TIFF Stack	https://doi.org/10.17602/M2/M22343	851.72 MB	0.072339259	0.072339259	0.072339259	105	462	48.51	2000	Duke Lemur Center CC BY-NC
DLC 6257f	*Eulemur albifrons*	Hands Zipped TIFF Stack	https://doi.org/10.17602/M2/M22345	510.06 MB	0.072339259	0.072339259	0.072339259	105	462	48.51	2000	Duke Lemur Center CC BY-NC
DLC 6257f	*Eulemur albifrons*	Feet Zipped TIFF Stack	https://doi.org/10.17602/M2/M22347	602.63 MB	0.072339259	0.072339259	0.072339259	105	462	48.51	2000	Duke Lemur Center CC BY-NC
DLC 6367m	*Eulemur albifrons*	Full Body Zipped TIFF Stack	https://doi.org/10.17602/M2/M36332	3.91 GB	0.097435437	0.097435437	0.097435437	105	462	48.51	1800	Duke Lemur Center CC BY-NC
DLC 6367m	*Eulemur albifrons*	Foot Zipped TIFF Stack	https://doi.org/10.17602/M2/M36712	840.05 MB	0.05718651	0.05718651	0.05718651	90	467	42.03	2000	Duke Lemur Center CC BY-NC
DLC 6367m	*Eulemur albifrons*	Hands Zipped TIFF Stack	https://doi.org/10.17602/M2/M36728	1.2 GB	0.047985416	0.047985416	0.047985416	85	529	44.97	2000	Duke Lemur Center CC BY-NC
DLC 6367m	*Eulemur albifrons*	Skull Zipped TIFF Stack	https://doi.org/10.17602/M2/M36734	1.92 GB	0.057632256	0.057632256	0.057632256	85	529	44.97	2000	Duke Lemur Center CC BY-NC
DLC 5776f	*Eulemur collaris*	Feet Zipped TIFF Stack	https://doi.org/10.17602/M2/M26212	1.53 GB	0.07017421	0.07017421	0.07017421	95	468	44.46	1800	Duke Lemur Center CC BY-NC
DLC 5776f	*Eulemur collaris*	Hands Zipped TIFF Stack	https://doi.org/10.17602/M2/M26214	293.1 MB	0.065335058	0.065335058	0.065335058	95	468	44.46	1800	Duke Lemur Center CC BY-NC
DLC 5776f	*Eulemur collaris*	Full Body Zipped TIFF Stack	https://doi.org/10.17602/M2/M26225	3.52 GB	0.095144369	0.095144369	0.095144369	110	450	49.5	1800	Duke Lemur Center CC BY-NC
DLC 5800m	*Eulemur collaris*	Full Body Zipped TIFF Stack	https://doi.org/10.17602/M2/M24487	6.95 GB	0.095557347	0.095557347	0.095557347	110	382	42.02	1800	Duke Lemur Center CC BY-NC
DLC 5800m	*Eulemur collaris*	Skull Zipped TIFF Stack	https://doi.org/10.17602/M2/M24498	1.85 GB	0.061797734	0.061797734	0.061797734	110	382	42.02	2000	Duke Lemur Center CC BY-NC
DLC 5800m	*Eulemur collaris*	Feet Zipped TIFF Stack	https://doi.org/10.17602/M2/M26038	1.22 GB	0.066519879	0.066519879	0.066519879	110	382	42.02	2000	Duke Lemur Center CC BY-NC
DLC 5800m	*Eulemur collaris*	Hands Zipped TIFF Stack	https://doi.org/10.17602/M2/M26040	841.5 MB	0.061797734	0.061797734	0.061797734	110	382	42.02	2000	Duke Lemur Center CC BY-NC
DLC 5919m	*Eulemur collaris*	Full Body Zipped TIFF Stack	https://doi.org/10.17602/M2/M26752	4.47 GB	0.10545484	0.10545484	0.10545484	105	414	43.47	1800	Duke Lemur Center CC BY-NC
DLC 5919m	*Eulemur collaris*	Feet Zipped TIFF Stack	https://doi.org/10.17602/M2/M26754	1.91 GB	0.072178498	0.072178498	0.072178498	105	414	43.47	1800	Duke Lemur Center CC BY-NC
DLC 5919m	*Eulemur collaris*	Hands Zipped TIFF Stack	https://doi.org/10.17602/M2/M26867	488.04 MB	0.062790163	0.062790163	0.062790163	105	414	43.47	1800	Duke Lemur Center CC BY-NC
DLC 5919m	*Eulemur collaris*	Skull Zipped TIFF Stack	https://doi.org/10.17602/M2/M26869	1.06 GB	0.06360478	0.06360478	0.06360478	105	414	43.47	1800	Duke Lemur Center CC BY-NC
DLC 5982f	*Eulemur collaris*	Full Body Zipped TIFF Stack	https://doi.org/10.17602/M2/M26218	4.07 GB	0.102257393	0.102257393	0.102257393	100	480	48	1800	Duke Lemur Center CC BY-NC
DLC 5982f	*Eulemur collaris*	Feet Zipped TIFF Stack	https://doi.org/10.17602/M2/M26220	1.14 GB	0.065846503	0.065846503	0.065846503	95	468	44.46	1800	Duke Lemur Center CC BY-NC
DLC 5982f	*Eulemur collaris*	Hands Zipped TIFF Stack	https://doi.org/10.17602/M2/M26222	936.29 MB	0.057301372	0.057301372	0.057301372	95	468	44.46	1800	Duke Lemur Center CC BY-NC
DLC 5982f	*Eulemur collaris*	Skull Zipped TIFF Stack	https://doi.org/10.17602/M2/M26224	1.4 GB	0.071583502	0.071583502	0.071583502	95	468	44.46	1800	Duke Lemur Center CC BY-NC
DLC 6225m	*Eulemur collaris*	Hand/Feet Zipped TIFF Stack	https://doi.org/10.17602/M2/M26228	606.09 MB	0.087543622	0.087543622	0.087543622	95	468	44.46	1800	Duke Lemur Center CC BY-NC
DLC 6225m	*Eulemur collaris*	Skull/hand Zipped TIFF Stack	https://doi.org/10.17602/M2/M26230	1.04 GB	0.069161154	0.069161154	0.069161154	105	424	44.52	1800	Duke Lemur Center CC BY-NC
DLC 6225m	*Eulemur collaris*	Full Body Zipped TIFF Stack	https://doi.org/10.17602/M2/M30681	5.35 GB	0.090182722	0.090182722	0.090182722	100	480	48	1800	Duke Lemur Center CC BY-NC
DLC 5948m	*Eulemur sanfordi*	Feet Zipped TIFF Stack	https://doi.org/10.17602/M2/M15433	1.55 GB	0.06218655	0.06218655	0.06218655	160	165	26.4	2000	Duke Lemur Center CC BY-NC
DLC 5948m	*Eulemur sanfordi*	Hand Zipped TIFF Stack	https://doi.org/10.17602/M2/M15438	786.7 MB	0.042960089	0.042960089	0.042960089	160	165	26.4	1800	Duke Lemur Center CC BY-NC
DLC 5948m	*Eulemur sanfordi*	Cranium Zipped TIFF Stack	https://doi.org/10.17602/M2/M15439	4.51 GB	0.046267938	0.046267938	0.046267938	160	165	16.5	1800	Duke Lemur Center CC BY-NC
DLC 5948m	*Eulemur sanfordi*	Hand Zipped TIFF Stack	https://doi.org/10.17602/M2/M15440	1010.02 MB	0.042960089	0.042960089	0.042960089	160	165	26.4	1800	Duke Lemur Center CC BY-NC
DLC 5948m	*Eulemur sanfordi*	Full Body Zipped TIFF Stack	https://doi.org/10.17602/M2/M15461	7.06 GB	0.108922616	0.108922616	0.108922616	160	165	26.4	1800	Duke Lemur Center CC BY-NC
DLC 1080m	*Galago moholi*	Skull Zipped TIFF Stack	https://doi.org/10.17602/M2/M14170	4.51 GB	0.025370026	0.025370026	0.025370026	190	129	24.51	2000	Duke Lemur Center CC BY-NC
DLC 1080m	*Galago moholi*	Foot Zipped TIFF Stack	https://doi.org/10.17602/M2/M14178	504.99 MB	0.025943503	0.025943503	0.025943503	190	129	24.51	2000	Duke Lemur Center CC BY-NC
DLC 1080m	*Galago moholi*	Hand Zipped TIFF Stack	https://doi.org/10.17602/M2/M14186	2.11 GB	0.016125461	0.016125461	0.016125461	190	110	20.9	2000	Duke Lemur Center CC BY-NC
DLC 1080m	*Galago moholi*	Full Body Zipped TIFF Stack	https://doi.org/10.17602/M2/M15953	6.91 GB	0.046641555	0.046641555	0.046641555	190	129	24.51	2000	Duke Lemur Center CC BY-NC
DLC 1087f	*Galago moholi*	Hand Zipped TIFF Stack	https://doi.org/10.17602/M2/M13869	5.81 GB	0.012471638	0.012471638	0.012471638	170	71	12.07	2000	Duke Lemur Center CC BY-NC
DLC 1087f	*Galago moholi*	Leg Zipped TIFF Stack	https://doi.org/10.17602/M2/M13871	5.72 GB	0.036122527	0.036122527	0.036122527	190	137	26.03	2000	Duke Lemur Center CC BY-NC
DLC 1087f	*Galago moholi*	Skull Zipped TIFF Stack	https://doi.org/10.17602/M2/M13875	6.78 GB	0.0218757	0.0218757	0.0218757	188	109	26.03	2000	Duke Lemur Center CC BY-NC
DLC 1087f	*Galago moholi*	Foot Zipped TIFF Stack	https://doi.org/10.17602/M2/M14194	849.09 MB	0.027454335	0.027454335	0.027454335	155	154	23.87	2000	Duke Lemur Center CC BY-NC
DLC 1087f	*Galago moholi*	Full Body Zipped TIFF Stack	https://doi.org/10.17602/M2/M16955	5.59 GB	0.036122527	0.036122527	0.036122527	190	137	26.03	2000	Duke Lemur Center CC BY-NC
DLC 2016f	*Galago moholi*	Feet Zipped TIFF Stack	https://doi.org/10.17602/M2/M14600	4.25 GB	0.023040427	0.023040427	0.023040427	170	129	21.93	2000	Duke Lemur Center CC BY-NC
DLC 2016f	*Galago moholi*	Hand Zipped TIFF Stack	https://doi.org/10.17602/M2/M14744	935.19 MB	0.018501708	0.018501708	0.018501708	170	102	17.34	2000	Duke Lemur Center CC BY-NC
DLC 2016f	*Galago moholi*	Hand(2) Zipped TIFF Stack	https://doi.org/10.17602/M2/M14748	763.51 MB	0.018501708	0.018501708	0.018501708	170	102	17.34	2000	Duke Lemur Center CC BY-NC
DLC 2016f	*Galago moholi*	Full Body Zipped TIFF Stack	https://doi.org/10.17602/M2/M25856	8.54 GB	0.047878649	0.047878649	0.047878649	170	168	28.56	2000	Duke Lemur Center CC BY-NC
DLC 2016f	*Galago moholi*	Skull Zipped TIFF Stack	https://doi.org/10.17602/M2/M33544	4.48 GB	0.021710116	0.021710116	0.021710116	170	123	20.91	2000	Duke Lemur Center CC BY-NC
DLC 2041m	*Galago moholi*	Foot Zipped TIFF Stack	https://doi.org/10.17602/M2/M14837	1.15 GB	0.026992276	0.026992276	0.026992276	170	153	26.01	2000	Duke Lemur Center CC BY-NC
DLC 2041m	*Galago moholi*	Hand Zipped TIFF Stack	https://doi.org/10.17602/M2/M14839	1.31 GB	0.017008841	0.017008841	0.017008841	160	100	16	2000	Duke Lemur Center CC BY-NC
DLC 2041m	*Galago moholi*	Hand(2) Zipped TIFF Stack	https://doi.org/10.17602/M2/M14840	1004.74 MB	0.017008841	0.017008841	0.017008841	160	100	16	2000	Duke Lemur Center CC BY-NC
DLC 2041m	*Galago moholi*	Skull Zipped TIFF Stack	https://doi.org/10.17602/M2/M14851	5.35 GB	0.02195541	0.02195541	0.02195541	170	120	20.4	2000	Duke Lemur Center CC BY-NC
DLC 2041m	*Galago moholi*	Full Body Zipped TIFF Stack	https://doi.org/10.17602/M2/M16397	6.54 GB	0.051666446	0.051666446	0.051666446	170	153	26.01	2000	Duke Lemur Center CC BY-NC
DLC 2061m	*Galago moholi*	Foot Zipped TIFF Stack	https://doi.org/10.17602/M2/M14846	1.17 GB	0.029418161	0.029418161	0.029418161	170	159	27.03	2000	Duke Lemur Center CC BY-NC
DLC 2061m	*Galago moholi*	Hand Zipped TIFF Stack	https://doi.org/10.17602/M2/M14858	2.78 GB	0.019379061	0.019379061	0.019379061	185	101	18.69	2000	Duke Lemur Center CC BY-NC
DLC 2061m	*Galago moholi*	Full Body Zipped TIFF Stack	https://doi.org/10.17602/M2/M16406	6.42 GB	0.050659675	0.050659675	0.050659675	170	171	29.07	2000	Duke Lemur Center CC BY-NC
DLC 3007f	*Galago moholi*	Foot Zipped TIFF Stack	https://doi.org/10.17602/M2/M15029	2.79 GB	0.019496443	0.019496443	0.019496443	150	110	16.5	2000	Duke Lemur Center CC BY-NC
DLC 3007f	*Galago moholi*	Hands Zipped TIFF Stack	https://doi.org/10.17602/M2/M15031	4.29 GB	0.019496443	0.019496443	0.019496443	170	94	15.98	2000	Duke Lemur Center CC BY-NC
DLC 3007f	*Galago moholi*	Full Body Zipped TIFF Stack	https://doi.org/10.17602/M2/M16919	1.11 GB	0.043311324	0.043311324	0.043311324	170	164	27.88	2000	Duke Lemur Center CC BY-NC
DLC 3123f	*Galago moholi*	Feet Zipped TIFF Stack	https://doi.org/10.17602/M2/M15063	2.29 GB	0.02990908	0.02990908	0.02990908	140	155	21.7	1800	Duke Lemur Center CC BY-NC
DLC 3123f	*Galago moholi*	Hands Zipped TIFF Stack	https://doi.org/10.17602/M2/M15065	2.8 GB	0.018665278	0.018665278	0.018665278	140	125	17.5	1800	Duke Lemur Center CC BY-NC
DLC 3123f	*Galago moholi*	Skull Zipped TIFF Stack	https://doi.org/10.17602/M2/M15067	4.51 GB	0.022479782	0.022479782	0.022479782	155	150	23.25	1800	Duke Lemur Center CC BY-NC
DLC 3123f	*Galago moholi*	Full Body Zipped TIFF Stack	https://doi.org/10.17602/M2/M16930	724.22 MB	0.044618849	0.044618849	0.044618849	145	166	24.07	1800	Duke Lemur Center CC BY-NC
DLC 3141f	*Galago moholi*	Hand Zipped TIFF Stack	https://doi.org/10.17602/M2/M15184	2.23 GB	0.014139019	0.014139019	0.014139019	170	81	13.77	2000	Duke Lemur Center CC BY-NC
DLC 3141f	*Galago moholi*	Feet Zipped TIFF Stack	https://doi.org/10.17602/M2/M15187	4.11 GB	0.024953157	0.024953157	0.024953157	160	147	23.52	2000	Duke Lemur Center CC BY-NC
DLC 3141f	*Galago moholi*	Skull Zipped TIFF Stack	https://doi.org/10.17602/M2/M15188	6.38 GB	0.021342928	0.021342928	0.021342928	170	120	20.4	2000	Duke Lemur Center CC BY-NC
DLC 3141f	*Galago moholi*	Full Body Zipped TIFF Stack	https://doi.org/10.17602/M2/M33668	5.05 GB	0.046546802	0.046546802	0.046546802	170	153	26.01	2000	Duke Lemur Center CC BY-NC
DLC 3143f	*Galago moholi*	Foot Zipped TIFF Stack	https://doi.org/10.17602/M2/M15191	1.24 GB	0.025652964	0.025652964	0.025652964	155	113	17.52	2000	Duke Lemur Center CC BY-NC
DLC 3143f	*Galago moholi*	Hand Zipped TIFF Stack	https://doi.org/10.17602/M2/M15192	797.56 MB	0.018613258	0.018613258	0.018613258	155	113	17.52	2000	Duke Lemur Center CC BY-NC
DLC 3143f	*Galago moholi*	Hand Zipped TIFF Stack	https://doi.org/10.17602/M2/M15195	710.81 MB	0.018613258	0.018613258	0.018613258	155	113	17.52	2000	Duke Lemur Center CC BY-NC
DLC 3143f	*Galago moholi*	Full Body Zipped TIFF Stack	https://doi.org/10.17602/M2/M33674	5.48 GB	0.046546802	0.046546802	0.046546802	170	153	26.01	2000	Duke Lemur Center CC BY-NC
DLC 3158f	*Galago moholi*	Foot Zipped TIFF Stack	https://doi.org/10.17602/M2/M15197	3.06 GB	0.023370048	0.023370048	0.023370048	190	129	24.51	2000	Duke Lemur Center CC BY-NC
DLC 3158f	*Galago moholi*	Hand Zipped TIFF Stack	https://doi.org/10.17602/M2/M15200	1.86 GB	0.015338421	0.015338421	0.015338421	190	129	24.51	2000	Duke Lemur Center CC BY-NC
DLC 3158f	*Galago moholi*	Hand Zipped TIFF Stack	https://doi.org/10.17602/M2/M15220	2.58 GB	0.015338421	0.015338421	0.015338421	190	129	24.51	2000	Duke Lemur Center CC BY-NC
DLC 3158f	*Galago moholi*	Skull Zipped TIFF Stack	https://doi.org/10.17602/M2/M15225	4.12 GB	0.024874931	0.024874931	0.024874931	190	129	24.51	2000	Duke Lemur Center CC BY-NC
DLC 3158f	*Galago moholi*	Full Body Zipped TIFF Stack	https://doi.org/10.17602/M2/M37275	5.71 GB	0.044695847	0.044695847	0.044695847	188	153	28.76	2000	Duke Lemur Center CC BY-NC
DLC 3185m	*Galago moholi*	Foot Zipped TIFF Stack	https://doi.org/10.17602/M2/M15199	955.8 MB	0.029039456	0.029039456	0.029039456	120	214	25.68	2000	Duke Lemur Center CC BY-NC
DLC 3185m	*Galago moholi*	Hand Zipped TIFF Stack	https://doi.org/10.17602/M2/M15201	2.08 GB	0.017496705	0.017496705	0.017496705	175	92	16.1	2000	Duke Lemur Center CC BY-NC
DLC 3185m	*Galago moholi*	Skull Zipped TIFF Stack	https://doi.org/10.17602/M2/M15223	2.11 GB	0.033769425	0.033769425	0.033769425	155	168	26.04	2000	Duke Lemur Center CC BY-NC
DLC 3185m	*Galago moholi*	Full Body Zipped TIFF Stack	https://doi.org/10.17602/M2/M16940	714.67 MB	0.056553539	0.056553539	0.056553539	155	168	26.04	1800	Duke Lemur Center CC BY-NC
DLC 3187m	*Galago moholi*	Foot Zipped TIFF Stack	https://doi.org/10.17602/M2/M15222	2.64 GB	0.023058226	0.023058226	0.023058226	135	137	18.5	2000	Duke Lemur Center CC BY-NC
DLC 3187m	*Galago moholi*	Hand Zipped TIFF Stack	https://doi.org/10.17602/M2/M15234	1.5 GB	0.01759561	0.01759561	0.01759561	135	137	18.23	1800	Duke Lemur Center CC BY-NC
DLC 3187m	*Galago moholi*	Skull Zipped TIFF Stack	https://doi.org/10.17602/M2/M15239	3.33 GB	0.024175918	0.024175918	0.024175918	135	137	18.23	2000	Duke Lemur Center CC BY-NC
DLC 3187m	*Galago moholi*	Full Body Zipped TIFF Stack	https://doi.org/10.17602/M2/M16942	834.23 MB	0.049657423	0.049657423	0.049657423	155	161	24.96	1800	Duke Lemur Center CC BY-NC
DLC 3190m	*Galago moholi*	Foot Zipped TIFF Stack	https://doi.org/10.17602/M2/M15236	1.94 GB	0.024251793	0.024251793	0.024251793	135	166	22.41	2000	Duke Lemur Center CC BY-NC
DLC 3190m	*Galago moholi*	Skull Zipped TIFF Stack	https://doi.org/10.17602/M2/M15241	5.43 GB	0.027059946	0.027059946	0.027059946	135	166	22.41	2000	Duke Lemur Center CC BY-NC
DLC 3190m	*Galago moholi*	Full Body Zipped TIFF Stack	https://doi.org/10.17602/M2/M16944	886.92 MB	0.053914855	0.053914855	0.053914855	135	191	25.79	1800	Duke Lemur Center CC BY-NC
DLC 3022m	*Galagoides demidovii*	Feet Zipped TIFF Stack	https://doi.org/10.17602/M2/M15033	2.42 GB	0.025294302	0.025294302	0.025294302	165	146	24.09	2000	Duke Lemur Center CC BY-NC
DLC 3022m	*Galagoides demidovii*	Hands Zipped TIFF Stack	https://doi.org/10.17602/M2/M15034	2.11 GB	0.016862897	0.016862897	0.016862897	165	106	17.49	2000	Duke Lemur Center CC BY-NC
DLC 3022m	*Galagoides demidovii*	Skull Zipped TIFF Stack	https://doi.org/10.17602/M2/M15035	2.6 GB	0.02517142	0.02517142	0.02517142	165	148	24.42	2000	Duke Lemur Center CC BY-NC
DLC 3022m	*Galagoides demidovii*	Full Body Zipped TIFF Stack	https://doi.org/10.17602/M2/M26216	2.11 GB	0.045630399	0.045630399	0.045630399	155	198	30.69	2000	Duke Lemur Center CC BY-NC
DLC 1302f	*Hapalemur griseus*	Lower Zipped TIFF Stack	https://doi.org/10.17602/M2/M13970	6.67 GB	0.088388361	0.088388361	0.088388361	125	277	34.63	1800	Duke Lemur Center CC BY-NC
DLC 1302f	*Hapalemur griseus*	Skull Zipped TIFF Stack	https://doi.org/10.17602/M2/M13977	7.15 GB	0.043946002	0.043946002	0.043946002	115	263	30.25	2000	Duke Lemur Center CC BY-NC
DLC 1302f	*Hapalemur griseus*	Hand Zipped TIFF Stack	https://doi.org/10.17602/M2/M14201	678.08 MB	0.043945989	0.043945989	0.043945989	115	263	30.25	2000	Duke Lemur Center CC BY-NC
DLC 1302f	*Hapalemur griseus*	Hands/Foot Zipped TIFF Stack	https://doi.org/10.17602/M2/M14207	3.79 GB	0.054910075	0.054910075	0.054910075	115	263	30.25	2000	Duke Lemur Center CC BY-NC
DLC 1302f	*Hapalemur griseus*	Upper Zipped TIFF Stack	https://doi.org/10.17602/M2/M14211	1.77 GB	0.088388361	0.088388361	0.088388361	125	277	34.63	1800	Duke Lemur Center CC BY-NC
DLC 1302f	*Hapalemur griseus*	Full Body Zipped TIFF Stack	https://doi.org/10.17602/M2/M15955	6.71 GB	0.088388361	0.088388361	0.088388361	125	277	34.63	1800	Duke Lemur Center CC BY-NC
DLC 1302f	*Hapalemur griseus*	Leg/Foot Zipped TIFF Stack	https://doi.org/10.17602/M2/M33807	1.65 GB	0.047857039	0.047857039	0.047857039	115	263	30.25	2000	Duke Lemur Center CC BY-NC
DLC 1311m	*Hapalemur griseus*	Full Body Zipped TIFF Stack	https://doi.org/10.17602/M2/M13973	5.07 GB	0.102538146	0.102538146	0.102538146	155	236	36.58	1800	Duke Lemur Center CC BY-NC
DLC 1311m	*Hapalemur griseus*	Hands Zipped TIFF Stack	https://doi.org/10.17602/M2/M13974	5.86 GB	0.032784853	0.032784853	0.032784853	130	241	31.33	2000	Duke Lemur Center CC BY-NC
DLC 1311m	*Hapalemur griseus*	Feet Zipped TIFF Stack	https://doi.org/10.17602/M2/M14214	4.37 GB	0.049337689	0.049337689	0.049337689	130	241	31.33	2000	Duke Lemur Center CC BY-NC
DLC 1313f	*Hapalemur griseus*	Foot Zipped TIFF Stack	https://doi.org/10.17602/M2/M14020	6.49 GB	0.037909437	0.037909437	0.037909437	150	234	35.1	2000	Duke Lemur Center CC BY-NC
DLC 1313f	*Hapalemur griseus*	Full Body Zipped TIFF Stack	https://doi.org/10.17602/M2/M14028	5.61 GB	0.092076518	0.092076518	0.092076518	150	220	33	1800	Duke Lemur Center CC BY-NC
DLC 1313f	*Hapalemur griseus*	Hand Zipped TIFF Stack	https://doi.org/10.17602/M2/M14228	2.92 GB	0.030636585	0.030636585	0.030636585	150	200	30	2000	Duke Lemur Center CC BY-NC
DLC 1317f	*Hapalemur griseus*	Foot Zipped TIFF Stack	https://doi.org/10.17602/M2/M14229	1.55 GB	0.044520918	0.044520918	0.044520918	120	276	33.12	2000	Duke Lemur Center CC BY-NC
DLC 1317f	*Hapalemur griseus*	Hand/Forearm Zipped TIFF Stack	https://doi.org/10.17602/M2/M14233	1.55 GB	0.054378938	0.054378938	0.054378938	115	278	31.97	2000	Duke Lemur Center CC BY-NC
DLC 1317f	*Hapalemur griseus*	Mid Zipped TIFF Stack	https://doi.org/10.17602/M2/M14235	1.55 GB	0.103797734	0.103797734	0.103797734	130	290	37.7	1800	Duke Lemur Center CC BY-NC
DLC 1317f	*Hapalemur griseus*	Skull Zipped TIFF Stack	https://doi.org/10.17602/M2/M14237	2.86 GB	0.04653291	0.04653291	0.04653291	120	294	35.28	2000	Duke Lemur Center CC BY-NC
DLC 1317f	*Hapalemur griseus*	Upper Zipped TIFF Stack	https://doi.org/10.17602/M2/M14241	1.67 GB	0.103797734	0.103797734	0.103797734	140	287	40.18	1800	Duke Lemur Center CC BY-NC
DLC 1317f	*Hapalemur griseus*	Lower Zipped TIFF Stack	https://doi.org/10.17602/M2/M16223	5.54 GB	0.103797734	0.103797734	0.103797734	130	290	37.7	1800	Duke Lemur Center CC BY-NC
DLC 1323f	*Hapalemur griseus*	Feet Zipped TIFF Stack	https://doi.org/10.17602/M2/M14246	2.49 GB	0.047259789	0.047259789	0.047259789	120	238	28.56	2000	Duke Lemur Center CC BY-NC
DLC 1323f	*Hapalemur griseus*	Hand Zipped TIFF Stack	https://doi.org/10.17602/M2/M14249	193.39 MB	0.0457347	0.0457347	0.0457347	120	238	28.56	2000	Duke Lemur Center CC BY-NC
DLC 1323f	*Hapalemur griseus*	Lower Zipped TIFF Stack	https://doi.org/10.17602/M2/M14259	3.26 GB	0.083882093	0.083882093	0.083882093	130	165	21.45	1800	Duke Lemur Center CC BY-NC
DLC 1323f	*Hapalemur griseus*	Upper Zipped TIFF Stack	https://doi.org/10.17602/M2/M14261	1.74 GB	0.083882093	0.083882093	0.083882093	130	265	34.45	1800	Duke Lemur Center CC BY-NC
DLC 1323f	*Hapalemur griseus*	Full Body Zipped TIFF Stack	https://doi.org/10.17602/M2/M15990	5.8 GB	0.083882093	0.083882093	0.083882093	130	265	34.45	1800	Duke Lemur Center CC BY-NC
DLC 1331f	*Hapalemur griseus*	Feet Zipped TIFF Stack	https://doi.org/10.17602/M2/M14263	2.42 GB	0.047602925	0.047602925	0.047602925	140	229	32.06	2000	Duke Lemur Center CC BY-NC
DLC 1331f	*Hapalemur griseus*	Hand Zipped TIFF Stack	https://doi.org/10.17602/M2/M14266	1.94 GB	0.036672633	0.036672633	0.036672633	145	245	35.53	2000	Duke Lemur Center CC BY-NC
DLC 1331f	*Hapalemur griseus*	Lower Zipped TIFF Stack	https://doi.org/10.17602/M2/M16442	3.29 GB	0.093477212	0.093477212	0.093477212	140	264	36.96	1800	Duke Lemur Center CC BY-NC
DLC 1331f	*Hapalemur griseus*	Upper Zipped TIFF Stack	https://doi.org/10.17602/M2/M16664	5.47 GB	0.093477212	0.093477212	0.093477212	140	264	39.96	1800	Duke Lemur Center CC BY-NC
DLC 1333m	*Hapalemur griseus*	Foot Zipped TIFF Stack	https://doi.org/10.17602/M2/M14281	941.63 MB	0.050929051	0.050929051	0.050929051	120	255	30.6	2000	Duke Lemur Center CC BY-NC
DLC 1333m	*Hapalemur griseus*	Hand Zipped TIFF Stack	https://doi.org/10.17602/M2/M14282	528.76 MB	0.04425583	0.04425583	0.04425583	120	255	30.6	2000	Duke Lemur Center CC BY-NC
DLC 1333m	*Hapalemur griseus*	Lower Zipped TIFF Stack	https://doi.org/10.17602/M2/M14288	2.29 GB	0.108927906	0.108927906	0.108927906	125	328	41	1800	Duke Lemur Center CC BY-NC
DLC 1333m	*Hapalemur griseus*	Skull Zipped TIFF Stack	https://doi.org/10.17602/M2/M14289	3.56 GB	0.04425583	0.04425583	0.04425583	120	255	30.6	2000	Duke Lemur Center CC BY-NC
DLC 1333m	*Hapalemur griseus*	Upper Zipped TIFF Stack	https://doi.org/10.17602/M2/M14290	2.95 GB	0.108927906	0.108927906	0.108927906	125	328	41	1800	Duke Lemur Center CC BY-NC
DLC 1333m	*Hapalemur griseus*	Full Body Zipped TIFF Stack	https://doi.org/10.17602/M2/M15991	5.72 GB	0.108927906	0.108927906	0.108927906	125	328	41	1800	Duke Lemur Center CC BY-NC
DLC 1337f	*Hapalemur griseus*	Feet Zipped TIFF Stack	https://doi.org/10.17602/M2/M14304	1.36 GB	0.055972494	0.055972494	0.055972494	115	279	32.09	2000	Duke Lemur Center CC BY-NC
DLC 1337f	*Hapalemur griseus*	Hands Zipped TIFF Stack	https://doi.org/10.17602/M2/M14309	2.42 GB	0.038098533	0.038098533	0.038098533	145	238	34.51	2000	Duke Lemur Center CC BY-NC
DLC 1337f	*Hapalemur griseus*	Skull Zipped TIFF Stack	https://doi.org/10.17602/M2/M14312	3 GB	0.051481672	0.051481672	0.051481672	130	238	30.94	2000	Duke Lemur Center CC BY-NC
DLC 1337f	*Hapalemur griseus*	Full Body Zipped TIFF Stack	https://doi.org/10.17602/M2/M35412	3.47 GB	0.093004674	0.093004674	0.093004674	155	232	35.96	1800	Duke Lemur Center CC BY-NC
DLC 1353f	*Hapalemur griseus*	Feet Zipped TIFF Stack	https://doi.org/10.17602/M2/M14315	2.42 GB	0.047832448	0.047832448	0.047832448	120	280	33.6	2000	Duke Lemur Center CC BY-NC
DLC 1353f	*Hapalemur griseus*	Hands Zipped TIFF Stack	https://doi.org/10.17602/M2/M14322	1.04 GB	0.047655281	0.047655281	0.047655281	120	280	33.6	2000	Duke Lemur Center CC BY-NC
DLC 1353f	*Hapalemur griseus*	Full Body Zipped TIFF Stack	https://doi.org/10.17602/M2/M35407	3.44 GB	0.099821776	0.099821776	0.099821776	145	245	35.53	2000	Duke Lemur Center CC BY-NC
DLC 1354m	*Hapalemur griseus*	Feet Zipped TIFF Stack	https://doi.org/10.17602/M2/M14324	2.47 GB	0.051356088	0.051356088	0.051356088	110	255	28.05	2000	Duke Lemur Center CC BY-NC
DLC 1354m	*Hapalemur griseus*	Hands Zipped TIFF Stack	https://doi.org/10.17602/M2/M14333	1.11 GB	0.051598269	0.051598269	0.051598269	110	255	28.05	2000	Duke Lemur Center CC BY-NC
DLC 1354m	*Hapalemur griseus*	Full Body Zipped TIFF Stack	https://doi.org/10.17602/M2/M35409	4 GB	0.093332745	0.093332745	0.093332745	145	269	39.01	2000	Duke Lemur Center CC BY-NC
DLC 1359m	*Hapalemur griseus*	Foot Zipped TIFF Stack	https://doi.org/10.17602/M2/M14334	1.33 GB	0.048827916	0.048827916	0.048827916	115	243	27.95	2000	Duke Lemur Center CC BY-NC
DLC 1359m	*Hapalemur griseus*	Hand Zipped TIFF Stack	https://doi.org/10.17602/M2/M14343	1.33 GB	0.04253133	0.04253133	0.04253133	115	243	27.95	2000	Duke Lemur Center CC BY-NC
DLC 1359m	*Hapalemur griseus*	Skull Zipped TIFF Stack	https://doi.org/10.17602/M2/M14345	1.89 GB	0.04468653	0.04468653	0.04468653	115	243	27.95	2000	Duke Lemur Center CC BY-NC
DLC 1359m	*Hapalemur griseus*	Full Body Zipped TIFF Stack	https://doi.org/10.17602/M2/M15993	7.08 GB	0.092646509	0.092646509	0.092646509	130	292	37.96	1800	Duke Lemur Center CC BY-NC
DLC 1360f	*Hapalemur griseus*	Feet Zipped TIFF Stack	https://doi.org/10.17602/M2/M14352	2.25 GB	0.045164652	0.045164652	0.045164652	120	255	30.6	2000	Duke Lemur Center CC BY-NC
DLC 1360f	*Hapalemur griseus*	Hands Zipped TIFF Stack	https://doi.org/10.17602/M2/M14358	3.79 GB	0.040284164	0.040284164	0.040284164	120	255	30.6	2000	Duke Lemur Center CC BY-NC
DLC 1360f	*Hapalemur griseus*	Full Body Zipped TIFF Stack	https://doi.org/10.17602/M2/M14359	3.86 GB	0.108927906	0.108927906	0.108927906	125	328	41	1800	Duke Lemur Center CC BY-NC
DLC 1360f	*Hapalemur griseus*	Skull Zipped TIFF Stack	https://doi.org/10.17602/M2/M14363	4.75 GB	0.041552003	0.041552003	0.041552003	120	255	28.05	2000	Duke Lemur Center CC BY-NC
DLC 1367f	*Hapalemur griseus*	Foot Zipped TIFF Stack	https://doi.org/10.17602/M2/M14365	1.06 GB	0.051249679	0.051249679	0.051249679	125	277	34.63	2000	Duke Lemur Center CC BY-NC
DLC 1367f	*Hapalemur griseus*	Hands Zipped TIFF Stack	https://doi.org/10.17602/M2/M14369	2.55 GB	0.049727984	0.049727984	0.049727984	125	277	34.63	2000	Duke Lemur Center CC BY-NC
DLC 1367f	*Hapalemur griseus*	Skull Zipped TIFF Stack	https://doi.org/10.17602/M2/M14372	2.92 GB	0.044953998	0.044953998	0.044953998	130	266	34.58	2000	Duke Lemur Center CC BY-NC
DLC 1367f	*Hapalemur griseus*	Upper Zipped TIFF Stack	https://doi.org/10.17602/M2/M16121	3.5 GB	0.101909123	0.101909123	0.101909123	140	282	39.48	1800	Duke Lemur Center CC BY-NC
DLC 1367f	*Hapalemur griseus*	Lower Zipped TIFF Stack	https://doi.org/10.17602/M2/M16122	3.84 GB	0.108830117	0.108830117	0.108830117	125	277	34.63	1800	Duke Lemur Center CC BY-NC
DLC 5977m	*Lemur catta*	Foot Zipped TIFF Stack	https://doi.org/10.17602/M2/M36742	822.81 MB	0.065080784	0.065080784	0.065080784	100	443	44.3	2000	Duke Lemur Center CC BY-NC
DLC 5977m	*Lemur catta*	Hand Zipped TIFF Stack	https://doi.org/10.17602/M2/M36744	367.49 MB	0.059766557	0.059766557	0.059766557	100	443	44.4	2000	Duke Lemur Center CC BY-NC
DLC 5977m	*Lemur catta*	Trunk Zipped TIFF Stack	https://doi.org/10.17602/M2/M38031	3.2 GB	0.110083237	0.110083237	0.110083237	110	420	46.2	1800	Duke Lemur Center CC BY-NC
DLC 5977m	*Lemur catta*	Lower Zipped TIFF Stack	https://doi.org/10.17602/M2/M38037	921.89 MB	0.110083237	0.110083237	0.110083237	110	420	46.2	1800	Duke Lemur Center CC BY-NC
DLC 6143m	*Lemur catta*	Foot Zipped TIFF Stack	https://doi.org/10.17602/M2/M34548	1.55 GB	0.073608287	0.073608287	0.073608287	135	334	45.09	2000	Duke Lemur Center CC BY-NC
DLC 6143m	*Lemur catta*	Skull Zipped TIFF Stack	https://doi.org/10.17602/M2/M36748	1.22 GB	0.069920294	0.069920294	0.069920294	135	334	45.09	2000	Duke Lemur Center CC BY-NC
DLC 6143m	*Lemur catta*	Hand/Forearm Zipped TIFF Stack	https://doi.org/10.17602/M2/M36767	1.4 GB	0.064154781	0.064154781	0.064154781	135	334	45.09	2000	Duke Lemur Center CC BY-NC
DLC 6143m	*Lemur catta*	Trunk Zipped TIFF Stack	https://doi.org/10.17602/M2/M37329	6.79 GB	0.095492706	0.095492706	0.095492706	135	334	45.09	2000	Duke Lemur Center CC BY-NC
DLC 6143m	*Lemur catta*	Lower Zipped TIFF Stack	https://doi.org/10.17602/M2/M37390	3.76 GB	0.095492594	0.095492594	0.095492594	135	334	45.09	2000	Duke Lemur Center CC BY-NC
DLC 6276f	*Lemur catta*	Upper Trunk Zipped TIFF Stack	https://doi.org/10.17602/M2/M18409	4.27 GB	0.095073804	0.095073804	0.095073804	135	334	45.09	2000	Duke Lemur Center CC BY-NC
DLC 6276f	*Lemur catta*	Lower Trunk Zipped TIFF Stack	https://doi.org/10.17602/M2/M18426	3.02 GB	0.095073804	0.095073804	0.095073804	135	334	45.09	2000	Duke Lemur Center CC BY-NC
DLC 6276f	*Lemur catta*	Skull Zipped TIFF Stack	https://doi.org/10.17602/M2/M36776	639.89 MB	0.074961379	0.074961379	0.074961379	135	334	45.09	2000	Duke Lemur Center CC BY-NC
DLC 6276f	*Lemur catta*	Feet Zipped TIFF Stack	https://doi.org/10.17602/M2/M36787	419.54 MB	0.084363788	0.084363788	0.084363788	135	334	45.09	2000	Duke Lemur Center CC BY-NC
DLC 6276f	*Lemur catta*	Hands Zipped TIFF Stack	https://doi.org/10.17602/M2/M36793	508.19 MB	0.076436751	0.076436751	0.076436751	135	334	45.09	2000	Duke Lemur Center CC BY-NC
DLC 6276f	*Lemur catta*	Full Body Zipped TIFF Stack	https://doi.org/10.17602/M2/M37408	6.41 GB	0.095073804	0.095073804	0.095073804	135	334	45.09	2000	Duke Lemur Center CC BY-NC
DLC 6808m	*Lemur catta*	Feet Zipped TIFF Stack	https://doi.org/10.17602/M2/M31773	1.71 GB	0.05675124	0.05675124	0.05675124	100	400	40	2000	Duke Lemur Center CC BY-NC
DLC 6808m	*Lemur catta*	Hand Zipped TIFF Stack	https://doi.org/10.17602/M2/M31775	360.6 MB	0.052849662	0.052849662	0.052849662	100	400	40	2000	Duke Lemur Center CC BY-NC
DLC 6808m	*Lemur catta*	Hand Zipped TIFF Stack	https://doi.org/10.17602/M2/M31777	207.96 MB	0.052849662	0.052849662	0.052849662	100	400	40	2000	Duke Lemur Center CC BY-NC
DLC 6808m	*Lemur catta*	Skull Zipped TIFF Stack	https://doi.org/10.17602/M2/M31779	933.54 MB	0.058450068	0.058450068	0.058450068	100	317	31.7	2000	Duke Lemur Center CC BY-NC
DLC 6808m	*Lemur catta*	Full Body Zipped TIFF Stack	https://doi.org/10.17602/M2/M32065	5.95 GB	0.110047594	0.110047594	0.110047594	120	389	46.68	2000	Duke Lemur Center CC BY-NC
DLC 6848m	*Lemur catta*	Full Body Zipped TIFF Stack	https://doi.org/10.17602/M2/M20293	3.47 GB	0.110195689	0.110195689	0.110195689	110	420	46.2	1800	Duke Lemur Center CC BY-NC
DLC 6848m	*Lemur catta*	Skull Zipped TIFF Stack	https://doi.org/10.17602/M2/M20309	1.1 GB	0.074588589	0.074588589	0.074588589	100	400	40	2000	Duke Lemur Center CC BY-NC
DLC 6848m	*Lemur catta*	Hand Zipped TIFF Stack	https://doi.org/10.17602/M2/M20311	429.51 MB	0.055105228	0.055105228	0.055105228	100	400	40	2000	Duke Lemur Center CC BY-NC
DLC 6848m	*Lemur catta*	Foot Zipped TIFF Stack	https://doi.org/10.17602/M2/M20313	504.25 MB	0.07286676	0.07286676	0.07286676	100	400	40	2000	Duke Lemur Center CC BY-NC
DLC 6862m	*Lemur catta*	Foot Zipped TIFF Stack	https://doi.org/10.17602/M2/M36799	679.48 MB	0.070695929	0.070695929	0.070695929	120	363	43.56	2000	Duke Lemur Center CC BY-NC
DLC 6862m	*Lemur catta*	Skull Zipped TIFF Stack	https://doi.org/10.17602/M2/M37266	741.68 MB	0.069929034	0.069929034	0.069929034	120	363	43.56	2000	Duke Lemur Center CC BY-NC
DLC 6862m	*Lemur catta*	Arm/Forearm Zipped TIFF Stack	https://doi.org/10.17602/M2/M37268	615.28 MB	0.074184626	0.074184626	0.074184626	120	163	19.56	2000	Duke Lemur Center CC BY-NC
DLC 6862m	*Lemur catta*	Hand Zipped TIFF Stack	https://doi.org/10.17602/M2/M37270	149.02 MB	0.074184626	0.074184626	0.074184626	120	363	43.56	2000	Duke Lemur Center CC BY-NC
DLC 6862m	*Lemur catta*	Trunk Zipped TIFF Stack	https://doi.org/10.17602/M2/M38007	4.4 GB	0.089312486	0.089312486	0.089312486	120	363	43.56	1800	Duke Lemur Center CC BY-NC
DLC 6862m	*Lemur catta*	Hind/Limb Zipped TIFF Stack	https://doi.org/10.17602/M2/M38009	2.54 GB	0.089312486	0.089312486	0.089312486	120	363	43.56	1800	Duke Lemur Center CC BY-NC
DLC 6862m	*Lemur catta*	Leg Zipped TIFF Stack	https://doi.org/10.17602/M2/M38011	1.17 GB	0.089312486	0.089312486	0.089312486	120	363	43.56	1800	Duke Lemur Center CC BY-NC
DLC 7142f	*Lemur catta*	Hands Zipped TIFF Stack	https://doi.org/10.17602/M2/M20926	1.22 GB	0.067883313	0.067883313	0.067883313	135	334	45.09	2000	Duke Lemur Center CC BY-NC
DLC 7142f	*Lemur catta*	Skull Zipped TIFF Stack	https://doi.org/10.17602/M2/M20928	538.44 MB	0.067883313	0.067883313	0.067883313	135	334	45.09	2000	Duke Lemur Center CC BY-NC
DLC 7142f	*Lemur catta*	Feet Zipped TIFF Stack	https://doi.org/10.17602/M2/M20930	498.62 MB	0.07595484	0.07595484	0.07595484	135	334	45.09	2000	Duke Lemur Center CC BY-NC
DLC 7142f	*Lemur catta*	Full Body Zipped TIFF Stack	https://doi.org/10.17602/M2/M32220	5.22 GB	0.089312486	0.089312486	0.089312486	120	363	43.56	1800	Duke Lemur Center CC BY-NC
DLC 1902f	*Loris tardigradus*	Full Body Zipped TIFF Stack	https://doi.org/10.17602/M2/M39783	889.58 MB	0.069091707	0.069091707	0.069091707	150	183	27.45	2000	Duke Lemur Center CC BY-NC
DLC 1902f	*Loris tardigradus*	Foot Zipped TIFF Stack	https://doi.org/10.17602/M2/M39785	345.09 MB	0.02812899	0.02812899	0.02812899	160	135	21.6	2000	Duke Lemur Center CC BY-NC
DLC 1902f	*Loris tardigradus*	Forelimbs Zipped TIFF Stack	https://doi.org/10.17602/M2/M39788	1.81 GB	0.039654193	0.039654193	0.039654193	150	149	22.35	2000	Duke Lemur Center CC BY-NC
DLC 1902f	*Loris tardigradus*	Hands Zipped TIFF Stack	https://doi.org/10.17602/M2/M39790	616.28 MB	0.028128993	0.028128993	0.028128993	160	135	21.6	2000	Duke Lemur Center CC BY-NC
DLC 1902f	*Loris tardigradus*	Hindlimbs Zipped TIFF Stack	https://doi.org/10.17602/M2/M39792	1.03 GB	0.044697583	0.044697583	0.044697583	150	149	22.35	2000	Duke Lemur Center CC BY-NC
DLC 1902f	*Loris tardigradus*	Skull Zipped TIFF Stack	https://doi.org/10.17602/M2/M39795	1.72 GB	0.030086279	0.030086279	0.030086279	155	134	20.77	2000	Duke Lemur Center CC BY-NC
DLC 1918m	*Loris tardigradus*	Hand/Feet Zipped TIFF Stack	https://doi.org/10.17602/M2/M14400	3.25 GB	0.02294362	0.02294362	0.02294362	155	120	18.6	2000	Duke Lemur Center CC BY-NC
DLC 1918m	*Loris tardigradus*	Skull Zipped TIFF Stack	https://doi.org/10.17602/M2/M16234	5.91 GB	0.026180163	0.026180163	0.026180163	155	120	18.6	2000	Duke Lemur Center CC BY-NC
DLC 1918m	*Loris tardigradus*	Full Body Zipped TIFF Stack	https://doi.org/10.17602/M2/M25554	5.92 GB	0.048001297	0.048001297	0.048001297	155	120	18.6	2000	Duke Lemur Center CC BY-NC
DLC 1992m	*Loris tardigradus*	Foot Zipped TIFF Stack	https://doi.org/10.17602/M2/M14402	3.25 GB	0.024378102	0.024378102	0.024378102	155	120	18.6	2000	Duke Lemur Center CC BY-NC
DLC 1992m	*Loris tardigradus*	Hand Zipped TIFF Stack	https://doi.org/10.17602/M2/M14475	263.17 MB	0.035715047	0.035715047	0.035715047	155	120	18.6	2000	Duke Lemur Center CC BY-NC
DLC 1992m	*Loris tardigradus*	Skull Zipped TIFF Stack	https://doi.org/10.17602/M2/M14477	3.11 GB	0.035715047	0.035715047	0.035715047	155	120	18.6	2000	Duke Lemur Center CC BY-NC
DLC 1992m	*Loris tardigradus*	Full Body Zipped TIFF Stack	https://doi.org/10.17602/M2/M16396	7.63 GB	0.049314659	0.049314659	0.049314659	155	120	18.6	2000	Duke Lemur Center CC BY-NC
DLC 2930m	*Loris tardigradus*	Foot Zipped TIFF Stack	https://doi.org/10.17602/M2/M15013	1.46 GB	0.031504329	0.031504329	0.031504329	155	120	18.6	2000	Duke Lemur Center CC BY-NC
DLC 2930m	*Loris tardigradus*	Hands Zipped TIFF Stack	https://doi.org/10.17602/M2/M15014	2.26 GB	0.027718766	0.027718766	0.027718766	155	120	18.6	2000	Duke Lemur Center CC BY-NC
DLC 2930m	*Loris tardigradus*	Skull Zipped TIFF Stack	https://doi.org/10.17602/M2/M15016	3.29 GB	0.035230957	0.035230957	0.035230957	155	120	18.6	2000	Duke Lemur Center CC BY-NC
DLC 2930m	*Loris tardigradus*	Full Body Zipped TIFF Stack	https://doi.org/10.17602/M2/M16454	6.68 GB	0.07634972	0.07634972	0.07634972	155	120	18.6	2000	Duke Lemur Center CC BY-NC
DLC 977f	*Loris tardigradus*	Full Body Zipped TIFF Stack	https://doi.org/10.17602/M2/M39766	1.24 GB	0.069091707	0.069091707	0.069091707	150	183	27.45	2000	Duke Lemur Center CC BY-NC
DLC 977f	*Loris tardigradus*	Feet Zipped TIFF Stack	https://doi.org/10.17602/M2/M39772	1.04 GB	0.026325268	0.026325268	0.026325268	145	111	16.1	2000	Duke Lemur Center CC BY-NC
DLC 977f	*Loris tardigradus*	Forelimb Zipped TIFF Stack	https://doi.org/10.17602/M2/M39774	1.39 GB	0.040545581	0.040545581	0.040545581	160	125	20	2000	Duke Lemur Center CC BY-NC
DLC 977f	*Loris tardigradus*	Hands Zipped TIFF Stack	https://doi.org/10.17602/M2/M39776	492.38 MB	0.028736417	0.028736417	0.028736417	160	135	21.6	2000	Duke Lemur Center CC BY-NC
DLC 977f	*Loris tardigradus*	Hindlimb Zipped TIFF Stack	https://doi.org/10.17602/M2/M39779	1.4 GB	0.040545581	0.040545581	0.040545581	160	125	20	2000	Duke Lemur Center CC BY-NC
DLC 977f	*Loris tardigradus*	Skull Zipped TIFF Stack	https://doi.org/10.17602/M2/M39781	1.52 GB	0.032464791	0.032464791	0.032464791	160	102	16.32	2000	Duke Lemur Center CC BY-NC
DLC 1812f	*Microcebus murinus*	Hand Zipped TIFF Stack	https://doi.org/10.17602/M2/M14787	260.46 MB	0.018870898	0.018870898	0.018870898	85	214	18.19	2000	Duke Lemur Center CC BY-NC
DLC 1812f	*Microcebus murinus*	Hand Zipped TIFF Stack	https://doi.org/10.17602/M2/M14833	94.64 MB	0.023938105	0.023938105	0.023938105	85	279	23.72	2000	Duke Lemur Center CC BY-NC
DLC 1812f	*Microcebus murinus*	Feet Zipped TIFF Stack	https://doi.org/10.17602/M2/M14835	1.06 GB	0.023938105	0.023938105	0.023938105	85	279	23.72	2000	Duke Lemur Center CC BY-NC
DLC 1812f	*Microcebus murinus*	Full Body Zipped TIFF Stack	https://doi.org/10.17602/M2/M16324	2.95 GB	0.040532459	0.040532459	0.040532459	85	419	35.62	1800	Duke Lemur Center CC BY-NC
DLC 1830f	*Microcebus murinus*	Foot Zipped TIFF Stack	https://doi.org/10.17602/M2/M15762	1.01 GB	0.019533198	0.019533198	0.019533198	105	177	18.59	2000	Duke Lemur Center CC BY-NC
DLC 1830f	*Microcebus murinus*	Hand Zipped TIFF Stack	https://doi.org/10.17602/M2/M15763	580.82 MB	0.022591772	0.022591772	0.022591772	100	219	21.9	2000	Duke Lemur Center CC BY-NC
DLC 1830f	*Microcebus murinus*	Hand Zipped TIFF Stack	https://doi.org/10.17602/M2/M15766	668.55 MB	0.022591772	0.022591772	0.022591772	100	219	21.9	2000	Duke Lemur Center CC BY-NC
DLC 1830f	*Microcebus murinus*	Full Body Zipped TIFF Stack	https://doi.org/10.17602/M2/M16235	3.11 GB	0.033495944	0.033495944	0.033495944	100	305	30.5	1800	Duke Lemur Center CC BY-NC
DLC 7003m	*Microcebus murinus*	Full Body Zipped TIFF Stack	https://doi.org/10.17602/M2/M34141	881.09 MB	0.051894952	0.051894952	0.051894952	90	283	25.47	1800	Duke Lemur Center CC BY-NC
DLC 7003m	*Microcebus murinus*	Hand/Foot Zipped TIFF Stack	https://doi.org/10.17602/M2/M34144	564.83 MB	0.026893973	0.026893973	0.026893973	90	214	19.26	2000	Duke Lemur Center CC BY-NC
DLC 7003m	*Microcebus murinus*	Skull Zipped TIFF Stack	https://doi.org/10.17602/M2/M34148	833.86 MB	0.024888759	0.024888759	0.024888759	90	214	19.26	2000	Duke Lemur Center CC BY-NC
DLC 7006m	*Microcebus murinus*	Full Body Zipped TIFF Stack	https://doi.org/10.17602/M2/M34100	915.65 MB	0.044273071	0.044273071	0.044273071	100	222	22.2	1800	Duke Lemur Center CC BY-NC
DLC 7006m	*Microcebus murinus*	Foot Zipped TIFF Stack	https://doi.org/10.17602/M2/M34102	658.76 MB	0.024626352	0.024626352	0.024626352	105	177	18.59	2000	Duke Lemur Center CC BY-NC
DLC 7006m	*Microcebus murinus*	Hand Zipped TIFF Stack	https://doi.org/10.17602/M2/M34104	335.28 MB	0.020458931	0.020458931	0.020458931	105	177	18.59	2000	Duke Lemur Center CC BY-NC
DLC 7006m	*Microcebus murinus*	Hand Zipped TIFF Stack	https://doi.org/10.17602/M2/M34106	214.88 MB	0.020458931	0.020458931	0.020458931	105	177	18.59	2000	Duke Lemur Center CC BY-NC
DLC 7011f	*Microcebus murinus*	Feet Zipped TIFF Stack	https://doi.org/10.17602/M2/M15453	1.01 GB	0.023221359	0.023221359	0.023221359	85	241	24.1	2000	Duke Lemur Center CC BY-NC
DLC 7011f	*Microcebus murinus*	Hands Zipped TIFF Stack	https://doi.org/10.17602/M2/M15455	711.03 MB	0.021238482	0.021238482	0.021238482	85	241	20.49	2000	Duke Lemur Center CC BY-NC
DLC 7011f	*Microcebus murinus*	Skull Zipped TIFF Stack	https://doi.org/10.17602/M2/M15460	2.04 GB	0.02131648	0.02131648	0.02131648	85	241	20.49	2000	Duke Lemur Center CC BY-NC
DLC 7011f	*Microcebus murinus*	Hand Zipped TIFF Stack	https://doi.org/10.17602/M2/M15476	163.23 MB	0.021238482	0.021238482	0.021238482	85	241	20.49	2000	Duke Lemur Center CC BY-NC
DLC 7011f	*Microcebus murinus*	Full Body Zipped TIFF Stack	https://doi.org/10.17602/M2/M33964	2.04 GB	0.040121231	0.040121231	0.040121231	85	419	35.62	1800	Duke Lemur Center CC BY-NC
DLC 7016m	*Microcebus murinus*	Foot Zipped TIFF Stack	https://doi.org/10.17602/M2/M15456	311.99 MB	0.026972702	0.026972702	0.026972702	90	272	24.48	2000	Duke Lemur Center CC BY-NC
DLC 7016m	*Microcebus murinus*	Skull Zipped TIFF Stack	https://doi.org/10.17602/M2/M15477	817.55 MB	0.027235521	0.027235521	0.027235521	90	272	24.48	2000	Duke Lemur Center CC BY-NC
DLC 7016m	*Microcebus murinus*	Hands Zipped TIFF Stack	https://doi.org/10.17602/M2/M15479	1.44 GB	0.027235521	0.027235521	0.027235521	90	272	24.48	2000	Duke Lemur Center CC BY-NC
DLC 7016m	*Microcebus murinus*	Full Body Zipped TIFF Stack	https://doi.org/10.17602/M2/M34021	2.75 GB	0.039243069	0.039243069	0.039243069	90	342	30.78	1800	Duke Lemur Center CC BY-NC
DLC 7017m	*Microcebus murinus*	Foot Zipped TIFF Stack	https://doi.org/10.17602/M2/M15714	439.62 MB	0.019744482	0.019744482	0.019744482	90	214	19.26	2000	Duke Lemur Center CC BY-NC
DLC 7017m	*Microcebus murinus*	Hand Zipped TIFF Stack	https://doi.org/10.17602/M2/M15715	288.36 MB	0.019744484	0.019744484	0.019744484	90	214	19.26	2000	Duke Lemur Center CC BY-NC
DLC 7017m	*Microcebus murinus*	Skull Zipped TIFF Stack	https://doi.org/10.17602/M2/M15720	2.03 GB	0.019744484	0.019744484	0.019744484	90	214	19.26	2000	Duke Lemur Center CC BY-NC
DLC 7017m	*Microcebus murinus*	Full Body Zipped TIFF Stack	https://doi.org/10.17602/M2/M15907	2.76 GB	0.038239431	0.038239431	0.038239431	90	342	30.78	1800	Duke Lemur Center CC BY-NC
DLC 7019m	*Microcebus murinus*	Full Body Zipped TIFF Stack	https://doi.org/10.17602/M2/M20937	670.23 MB	0.035296075	0.035296075	0.035296075	100	197	19.7	1800	Duke Lemur Center CC BY-NC
DLC 7019m	*Microcebus murinus*	Skull Zipped TIFF Stack	https://doi.org/10.17602/M2/M20939	1.29 GB	0.021320194	0.021320194	0.021320194	95	199	18.91	2000	Duke Lemur Center CC BY-NC
DLC 7019m	*Microcebus murinus*	Hand Zipped TIFF Stack	https://doi.org/10.17602/M2/M20941	228.17 MB	0.023636376	0.023636376	0.023636376	80	290	23.2	2000	Duke Lemur Center CC BY-NC
DLC 7019m	*Microcebus murinus*	Foot Zipped TIFF Stack	https://doi.org/10.17602/M2/M22060	314.44 MB	0.023878347	0.023878347	0.023878347	95	199	18.91	2000	Duke Lemur Center CC BY-NC
DLC 7021m	*Microcebus murinus*	Foot A Zipped TIFF Stack	https://doi.org/10.17602/M2/M15723	202.04 MB	0.022323428	0.022323428	0.022323428	95	216	20.52	2000	Duke Lemur Center CC BY-NC
DLC 7021m	*Microcebus murinus*	Foot B Zipped TIFF Stack	https://doi.org/10.17602/M2/M15727	314.48 MB	0.020491783	0.020491783	0.020491783	85	229	19.47	1800	Duke Lemur Center CC BY-NC
DLC 7021m	*Microcebus murinus*	Hand Zipped TIFF Stack	https://doi.org/10.17602/M2/M15730	278.8 MB	0.019185083	0.019185083	0.019185083	100	180	18	2000	Duke Lemur Center CC BY-NC
DLC 7021m	*Microcebus murinus*	Hand Zipped TIFF Stack	https://doi.org/10.17602/M2/M15731	229.43 MB	0.019185083	0.019185083	0.019185083	100	180	18	2000	Duke Lemur Center CC BY-NC
DLC 7021m	*Microcebus murinus*	Skull Zipped TIFF Stack	https://doi.org/10.17602/M2/M15733	2.38 GB	0.021313295	0.021313295	0.021313295	95	216	20.52	2000	Duke Lemur Center CC BY-NC
DLC 7021m	*Microcebus murinus*	Full Body Zipped TIFF Stack	https://doi.org/10.17602/M2/M15911	2.19 GB	0.038027935	0.038027935	0.038027935	85	413	35.11	1800	Duke Lemur Center CC BY-NC
DLC 7022f	*Microcebus murinus*	Full Body Zipped TIFF Stack	https://doi.org/10.17602/M2/M17127	17.13 MB	0.039805587	0.039805587	0.039805587	85	376	31.96	1800	Duke Lemur Center CC BY-NC
DLC 7024m	*Microcebus murinus*	Full Body Zipped TIFF Stack	https://doi.org/10.17602/M2/M17961	2.49 GB	0.044094801	0.044094801	0.044094801	100	272	27.2	1800	Duke Lemur Center CC BY-NC
DLC 7024m	*Microcebus murinus*	Feet Zipped TIFF Stack	https://doi.org/10.17602/M2/M26098	1013.63 MB	0.022291776	0.022291776	0.022291776	100	222	22.2	2000	Duke Lemur Center CC BY-NC
DLC 7024m	*Microcebus murinus*	Hand/Arm Zipped TIFF Stack	https://doi.org/10.17602/M2/M26099	1.48 GB	0.022863461	0.022863461	0.022863461	100	222	22.2	2000	Duke Lemur Center CC BY-NC
DLC 7024m	*Microcebus murinus*	Skull Zipped TIFF Stack	https://doi.org/10.17602/M2/M26102	1.15 GB	0.022863463	0.022863463	0.022863463	100	222	22.2	2000	Duke Lemur Center CC BY-NC
DLC 7025m	*Microcebus murinus*	Full Body Zipped TIFF Stack	https://doi.org/10.17602/M2/M17441	2.27 GB	0.046328414	0.046328414	0.046328414	85	376	31.96	1800	Duke Lemur Center CC BY-NC
DLC 7025m	*Microcebus murinus*	Foot Zipped TIFF Stack	https://doi.org/10.17602/M2/M34092	602.37 MB	0.024948237	0.024948237	0.024948237	85	271	23.04	2000	Duke Lemur Center CC BY-NC
DLC 7025m	*Microcebus murinus*	Foot Zipped TIFF Stack	https://doi.org/10.17602/M2/M34094	465.01 MB	0.024948237	0.024948237	0.024948237	85	271	23.04	2000	Duke Lemur Center CC BY-NC
DLC 7025m	*Microcebus murinus*	Hand Zipped TIFF Stack	https://doi.org/10.17602/M2/M34096	306.7 MB	0.024948237	0.024948237	0.024948237	85	271	23.04	2000	Duke Lemur Center CC BY-NC
DLC 7025m	*Microcebus murinus*	Hand Zipped TIFF Stack	https://doi.org/10.17602/M2/M34098	298.4 MB	0.024948237	0.024948237	0.024948237	85	271	23.04	2000	Duke Lemur Center CC BY-NC
DLC 7065m	*Microcebus murinus*	Full Body Zipped TIFF Stack	https://doi.org/10.17602/M2/M20932	375.35 MB	0.042540975	0.042540975	0.042540975	80	371	29.68	1800	Duke Lemur Center CC BY-NC
DLC 7065m	*Microcebus murinus*	Feet Zipped TIFF Stack	https://doi.org/10.17602/M2/M20934	846.4 MB	0.021827504	0.021827504	0.021827504	85	255	21.68	2000	Duke Lemur Center CC BY-NC
DLC 7065m	*Microcebus murinus*	Maxilla Zipped TIFF Stack	https://doi.org/10.17602/M2/M21458	172.05 MB	0.024335912	0.024335912	0.024335912	85	270	229.5	2000	Duke Lemur Center CC BY-NC
DLC 7065m	*Microcebus murinus*	Hand Zipped TIFF Stack	https://doi.org/10.17602/M2/M22080	159.37 MB	0.021827505	0.021827505	0.021827505	85	255	21.68	2000	Duke Lemur Center CC BY-NC
DLC 7065m	*Microcebus murinus*	Hand Zipped TIFF Stack	https://doi.org/10.17602/M2/M22082	206.75 MB	0.021827505	0.021827505	0.021827505	85	255	21.68	2000	Duke Lemur Center CC BY-NC
DLC 7065m	*Microcebus murinus*	Mandible Zipped TIFF Stack	https://doi.org/10.17602/M2/M22087	569.42 MB	0.021827505	0.021827505	0.021827505	85	255	21.68	2000	Duke Lemur Center CC BY-NC
DLC 845f	*Microcebus murinus*	Foot B Zipped TIFF Stack	https://doi.org/10.17602/M2/M15608	334.53 MB	0.021745628	0.021745628	0.021745628	90	215	19.35	2000	Duke Lemur Center CC BY-NC
DLC 845f	*Microcebus murinus*	Skull Zipped TIFF Stack	https://doi.org/10.17602/M2/M15612	1.76 GB	0.020519108	0.020519108	0.020519108	90	215	19.35	1800	Duke Lemur Center CC BY-NC
DLC 845f	*Microcebus murinus*	Full Body Zipped TIFF Stack	https://doi.org/10.17602/M2/M15902	2.47 GB	0.038239427	0.038239427	0.038239427	90	383	34.47	1800	Duke Lemur Center CC BY-NC
DLC 845f	*Microcebus murinus*	Hand Zipped TIFF Stack	https://doi.org/10.17602/M2/M16137	159.83 MB	0.020519106	0.020519106	0.020519106	90	215	19.35	2000	Duke Lemur Center CC BY-NC
DLC 845f	*Microcebus murinus*	Hand Zipped TIFF Stack	https://doi.org/10.17602/M2/M16140	182.08 MB	0.020519106	0.020519106	0.020519106	90	215	19.35	2000	Duke Lemur Center CC BY-NC
DLC 845f	*Microcebus murinus*	Foot Zipped TIFF Stack	https://doi.org/10.17602/M2/M16141	159.51 MB	0.020519106	0.020519106	0.020519106	90	215	19.35	2000	Duke Lemur Center CC BY-NC
DLC 893m	*Microcebus murinus*	Skull Zipped TIFF Stack	https://doi.org/10.17602/M2/M34257	1.19 GB	0.021885242	0.021885242	0.021885242	80	290	23.2	2000	Duke Lemur Center CC BY-NC
DLC 893m	*Microcebus murinus*	Full Body Zipped TIFF Stack	https://doi.org/10.17602/M2/M34259	2.19 GB	0.040855881	0.040855881	0.040855881	90	355	31.95	31.95	Duke Lemur Center CC BY-NC
DLC 893m	*Microcebus murinus*	Hand Zipped TIFF Stack	https://doi.org/10.17602/M2/M34261	146.22 MB	0.024257032	0.024257032	0.024257032	85	263	22.36	2000	Duke Lemur Center CC BY-NC
DLC 893m	*Microcebus murinus*	Hand Zipped TIFF Stack	https://doi.org/10.17602/M2/M34263	576.35 MB	0.024257032	0.024257032	0.024257032	85	263	22.36	2000	Duke Lemur Center CC BY-NC
DLC 893m	*Microcebus murinus*	Feet Zipped TIFF Stack	https://doi.org/10.17602/M2/M34265	496.63 MB	0.025659679	0.025659679	0.025659679	85	263	22.36	2000	Duke Lemur Center CC BY-NC
DLC NN_Mm01	*Microcebus murinus*	Full Body Zipped TIFF Stack	https://doi.org/10.17602/M2/M18378	2.78 GB	0.036884505	0.036884505	0.036884505	100	240	24	1800	Duke Lemur Center CC BY-NC
DLC NN_Mm01	*Microcebus murinus*	Foot Zipped TIFF Stack	https://doi.org/10.17602/M2/M34136	673.22 MB	0.024511045	0.024511045	0.024511045	100	210	21	2000	Duke Lemur Center CC BY-NC
DLC NN_Mm01	*Microcebus murinus*	Hand Zipped TIFF Stack	https://doi.org/10.17602/M2/M34138	459.36 MB	0.019862801	0.019862801	0.019862801	105	177	18.59	2000	Duke Lemur Center CC BY-NC
DLC NN_Mm02	*Microcebus murinus*	Full Body Zipped TIFF Stack	https://doi.org/10.17602/M2/M18383	1.75 GB	0.033327077	0.033327077	0.033327077	95	232	22.04	1800	Duke Lemur Center CC BY-NC
DLC NN_Mm02	*Microcebus murinus*	Feet Zipped TIFF Stack	https://doi.org/10.17602/M2/M34251	875.79 MB	0.022636365	0.022636365	0.022636365	80	275	22	2000	Duke Lemur Center CC BY-NC
DLC NN_Mm02	*Microcebus murinus*	Hands Zipped TIFF Stack	https://doi.org/10.17602/M2/M34253	662.74 MB	0.022636365	0.022636365	0.022636365	80	275	22	2000	Duke Lemur Center CC BY-NC
DLC NN_Mm02	*Microcebus murinus*	Skull Zipped TIFF Stack	https://doi.org/10.17602/M2/M34255	1.01 GB	0.02571173	0.02571173	0.02571173	80	290	23.2	2000	Duke Lemur Center CC BY-NC
DLC 2301f	*Mirza zaza*	Foot Zipped TIFF Stack	https://doi.org/10.17602/M2/M14856	594.37 MB	0.038561612	0.038561612	0.038561612	140	154	21.56	2000	Duke Lemur Center CC BY-NC
DLC 2301f	*Mirza zaza*	Calvarium Zipped TIFF Stack	https://doi.org/10.17602/M2/M14859	244.76 MB	0.038561612	0.038561612	0.038561612	140	154	21.56	2000	Duke Lemur Center CC BY-NC
DLC 2301f	*Mirza zaza*	Hand Zipped TIFF Stack	https://doi.org/10.17602/M2/M14864	129.57 MB	0.038561612	0.038561612	0.038561612	140	154	21.56	2000	Duke Lemur Center CC BY-NC
DLC 2301f	*Mirza zaza*	Skull Zipped TIFF Stack	https://doi.org/10.17602/M2/M14865	1.37 GB	0.038561612	0.038561612	0.038561612	140	154	21.56	2000	Duke Lemur Center CC BY-NC
DLC 2301f	*Mirza zaza*	Full Body Zipped TIFF Stack	https://doi.org/10.17602/M2/M16407	6.42 GB	0.067536809	0.067536809	0.067536809	140	154	21.56	1800	Duke Lemur Center CC BY-NC
DLC 2304m	*Mirza zaza*	Foot Zipped TIFF Stack	https://doi.org/10.17602/M2/M14871	1.55 GB	0.025382072	0.025382072	0.025382072	130	187	24.31	2000	Duke Lemur Center CC BY-NC
DLC 2304m	*Mirza zaza*	Hand Zipped TIFF Stack	https://doi.org/10.17602/M2/M14876	823.69 MB	0.025382072	0.025382072	0.025382072	130	187	24.31	2000	Duke Lemur Center CC BY-NC
DLC 2304m	*Mirza zaza*	Foot Zipped TIFF Stack	https://doi.org/10.17602/M2/M14877	1.17 GB	0.025382072	0.025382072	0.025382072	130	187	24.31	2000	Duke Lemur Center CC BY-NC
DLC 2304m	*Mirza zaza*	Skull Zipped TIFF Stack	https://doi.org/10.17602/M2/M14880	3.31 GB	0.029101968	0.029101968	0.029101968	120	186	22.32	2000	Duke Lemur Center CC BY-NC
DLC 2304m	*Mirza zaza*	Full Body Zipped TIFF Stack	https://doi.org/10.17602/M2/M16435	6.33 GB	0.061254796	0.061254796	0.061254796	120	260	31.2	1800	Duke Lemur Center CC BY-NC
DLC 2316m	*Mirza zaza*	Feet Zipped TIFF Stack	https://doi.org/10.17602/M2/M14885	3.37 GB	0.026818974	0.026818974	0.026818974	130	128	16.64	2000	Duke Lemur Center CC BY-NC
DLC 2316m	*Mirza zaza*	Skull Zipped TIFF Stack	https://doi.org/10.17602/M2/M14888	2.97 GB	0.026793329	0.026793329	0.026793329	150	130	19.5	2000	Duke Lemur Center CC BY-NC
DLC 2316m	*Mirza zaza*	Hand Zipped TIFF Stack	https://doi.org/10.17602/M2/M14890	1.46 GB	0.023880169	0.023880169	0.023880169	130	128	16.64	2000	Duke Lemur Center CC BY-NC
DLC 2316m	*Mirza zaza*	Lower Trunk Zipped TIFF Stack	https://doi.org/10.17602/M2/M19971	2.44 GB	0.049792167	0.049792167	0.049792167	130	169	21.97	1800	Duke Lemur Center CC BY-NC
DLC 2316m	*Mirza zaza*	Upper Body Zipped TIFF Stack	https://doi.org/10.17602/M2/M35403	3.23 GB	0.049792167	0.049792167	0.049792167	130	169	21.97	1800	Duke Lemur Center CC BY-NC
DLC 2322f	*Mirza zaza*	Hands Zipped TIFF Stack	https://doi.org/10.17602/M2/M14901	1.39 GB	0.027688136	0.027688136	0.027688136	130	204	26.52	2000	Duke Lemur Center CC BY-NC
DLC 2322f	*Mirza zaza*	Feet Zipped TIFF Stack	https://doi.org/10.17602/M2/M14904	2.58 GB	0.032836959	0.032836959	0.032836959	130	231	30.03	2000	Duke Lemur Center CC BY-NC
DLC 2322f	*Mirza zaza*	Full Body Zipped TIFF Stack	https://doi.org/10.17602/M2/M14907	4.89 GB	0.09669634	0.09669634	0.09669634	155	226	35.03	1800	Duke Lemur Center CC BY-NC
DLC 2322f	*Mirza zaza*	Skull Zipped TIFF Stack	https://doi.org/10.17602/M2/M14918	3.16 GB	0.033058789	0.033058789	0.033058789	130	231	30.03	2000	Duke Lemur Center CC BY-NC
DLC 315m	*Mirza zaza*	Foot Zipped TIFF Stack	https://doi.org/10.17602/M2/M15123	2.06 GB	0.027385578	0.027385578	0.027385578	170	157	26.69	2000	Duke Lemur Center CC BY-NC
DLC 315m	*Mirza zaza*	Hand Zipped TIFF Stack	https://doi.org/10.17602/M2/M15124	1.02 GB	0.027385578	0.027385578	0.027385578	170	157	26.69	2000	Duke Lemur Center CC BY-NC
DLC 315m	*Mirza zaza*	Full Body Zipped TIFF Stack	https://doi.org/10.17602/M2/M15125	5.63 GB	0.071125828	0.071125828	0.071125828	170	182	30.94	1800	Duke Lemur Center CC BY-NC
DLC 315m	*Mirza zaza*	Skull Zipped TIFF Stack	https://doi.org/10.17602/M2/M33799	3.66 GB	0.031418357	0.031418357	0.031418357	150	199	29.85	2000	Duke Lemur Center CC BY-NC
DLC 339f	*Mirza zaza*	Foot Zipped TIFF Stack	https://doi.org/10.17602/M2/M15150	1.42 GB	0.030469423	0.030469423	0.030469423	135	160	21.6	2000	Duke Lemur Center CC BY-NC
DLC 339f	*Mirza zaza*	Full Body Zipped TIFF Stack	https://doi.org/10.17602/M2/M15153	4.41 GB	0.070778213	0.070778213	0.070778213	135	241	32.54	1800	Duke Lemur Center CC BY-NC
DLC 339f	*Mirza zaza*	Foot Zipped TIFF Stack	https://doi.org/10.17602/M2/M15155	1.73 GB	0.030469423	0.030469423	0.030469423	135	160	21.6	2000	Duke Lemur Center CC BY-NC
DLC 339f	*Mirza zaza*	Hand Zipped TIFF Stack	https://doi.org/10.17602/M2/M15159	345.05 MB	0.031063354	0.031063354	0.031063354	135	160	21.6	2000	Duke Lemur Center CC BY-NC
DLC 339f	*Mirza zaza*	Hand Zipped TIFF Stack	https://doi.org/10.17602/M2/M15160	309.46 MB	0.031063354	0.031063354	0.031063354	135	160	21.6	2000	Duke Lemur Center CC BY-NC
DLC 340m	*Mirza zaza*	Hands Zipped TIFF Stack	https://doi.org/10.17602/M2/M15177	2.25 GB	0.021991465	0.021991465	0.021991465	155	134	20.77	2000	Duke Lemur Center CC BY-NC
DLC 340m	*Mirza zaza*	Feet Zipped TIFF Stack	https://doi.org/10.17602/M2/M15178	2.56 GB	0.027049243	0.027049243	0.027049243	155	134	20.77	2000	Duke Lemur Center CC BY-NC
DLC 340m	*Mirza zaza*	Full Body Zipped TIFF Stack	https://doi.org/10.17602/M2/M15899	4.45 GB	0.060477514	0.060477514	0.060477514	150	208	31.2	1800	Duke Lemur Center CC BY-NC
DLC 360m	*Mirza zaza*	Foot Zipped TIFF Stack	https://doi.org/10.17602/M2/M15175	1.45 GB	0.034495082	0.034495082	0.034495082	140	205	28.7	2000	Duke Lemur Center CC BY-NC
DLC 360m	*Mirza zaza*	Full Body Zipped TIFF Stack	https://doi.org/10.17602/M2/M15180	4.82 GB	0.073436119	0.073436119	0.073436119	130	231	30.03	2000	Duke Lemur Center CC BY-NC
DLC 360m	*Mirza zaza*	Skull Zipped TIFF Stack	https://doi.org/10.17602/M2/M15183	2.61 GB	0.034953907	0.034953907	0.034953907	140	220	30.8	2000	Duke Lemur Center CC BY-NC
DLC 373f	*Mirza zaza*	Full Body microCT Volume File	https://doi.org/10.17602/M2/M42257	1.56 GB	0.085649452	0.085649452	0.085649452	138	167	23.05	2000	Duke Lemur Center CC BY-NC
DLC 373f	*Mirza zaza*	Forelimb Zipped TIFF Stack	https://doi.org/10.17602/M2/M42258	4.13 GB	0.039029521	0.039029521	0.039029521	116	178	20.65	2000	Duke Lemur Center CC BY-NC
DLC 373f	*Mirza zaza*	Hindlimb microCT Volume File	https://doi.org/10.17602/M2/M42259	5.04 GB	0.049629849	0.049629849	0.049629849	131	166	21.75	2000	Duke Lemur Center CC BY-NC
DLC 373f	*Mirza zaza*	Skull microCT Volume File	https://doi.org/10.17602/M2/M42269	5.05 GB	0.030318779	0.030318779	0.030318779	179	59	10.56	2000	Duke Lemur Center CC BY-NC
DLC 1998f	*Nycticebus coucang*	Hand Zipped TIFF Stack	https://doi.org/10.17602/M2/M14595	4.76 GB	0.035238422	0.035238422	0.035238422	155	110	17.05	2000	Duke Lemur Center CC BY-NC
DLC 1998f	*Nycticebus coucang*	Skull Zipped TIFF Stack	https://doi.org/10.17602/M2/M14602	3.74 GB	0.041094869	0.041094869	0.041094869	155	110	17.05	2000	Duke Lemur Center CC BY-NC
DLC 1998f	*Nycticebus coucang*	Full Body Zipped TIFF Stack	https://doi.org/10.17602/M2/M14768	3.74 GB	0.106712222	0.106712222	0.106712222	155	110	17.05	2000	Duke Lemur Center CC BY-NC
DLC 1998f	*Nycticebus coucang*	Feet Zipped TIFF Stack	https://doi.org/10.17602/M2/M26668	3.97 GB	0.035397302	0.035397302	0.035397302	155	110	17.05	2000	Duke Lemur Center CC BY-NC
DLC 993m	*Nycticebus coucang*	Feet Zipped TIFF Stack	https://doi.org/10.17602/M2/M39714	2.03 GB	0.033481211	0.033481211	0.033481211	150	146	21.9	2000	Duke Lemur Center CC BY-NC
DLC 993m	*Nycticebus coucang*	Full Body Zipped TIFF Stack	https://doi.org/10.17602/M2/M39724	2.31 GB	0.080906598	0.080906598	0.080906598	145	166	24.07	2000	Duke Lemur Center CC BY-NC
DLC 993m	*Nycticebus coucang*	Skull Zipped TIFF Stack	https://doi.org/10.17602/M2/M39726	2.15 GB	0.039299007	0.039299007	0.039299007	145	173	25.09	2000	Duke Lemur Center CC BY-NC
DLC 993m	*Nycticebus coucang*	Hindlimbs Zipped TIFF Stack	https://doi.org/10.17602/M2/M39825	1.77 GB	0.053029843	0.053029843	0.053029843	160	193	30.88	2000	Duke Lemur Center CC BY-NC
DLC 993m	*Nycticebus coucang*	Forelimbs Zipped TIFF Stack	https://doi.org/10.17602/M2/M39827	1.86 GB	0.053029843	0.053029843	0.053029843	160	208	33.28	2000	Duke Lemur Center CC BY-NC
DLC 993m	*Nycticebus coucang*	Hands Zipped TIFF Stack	https://doi.org/10.17602/M2/M39829	1.06 GB	0.037518788	0.037518788	0.037518788	150	160	24	2000	Duke Lemur Center CC BY-NC
DLC 2901f	*Nycticebus pygmaeus*	Full Body Zipped TIFF Stack	https://doi.org/10.17602/M2/M14935	3.27 GB	0.077386118	0.077386118	0.077386118	190	85	16.15	2000	Duke Lemur Center CC BY-NC
DLC 2901f	*Nycticebus pygmaeus*	Skull Zipped TIFF Stack	https://doi.org/10.17602/M2/M14936	3.38 GB	0.035473477	0.035473477	0.035473477	155	110	17.05	2000	Duke Lemur Center CC BY-NC
DLC 2901f	*Nycticebus pygmaeus*	Hand Zipped TIFF Stack	https://doi.org/10.17602/M2/M14937	1.58 GB	0.037053335	0.037053335	0.037053335	190	85	16.15	2000	Duke Lemur Center CC BY-NC
DLC 2901f	*Nycticebus pygmaeus*	Foot Zipped TIFF Stack	https://doi.org/10.17602/M2/M14955	3.22 GB	0.034817457	0.034817457	0.034817457	185	81	14.99	2000	Duke Lemur Center CC BY-NC
DLC 2921m	*Nycticebus pygmaeus*	Foot Zipped TIFF Stack	https://doi.org/10.17602/M2/M14954	2.05 GB	0.027394786	0.027394786	0.027394786	150	100	15	2000	Duke Lemur Center CC BY-NC
DLC 2921m	*Nycticebus pygmaeus*	Hand Zipped TIFF Stack	https://doi.org/10.17602/M2/M14959	1.98 GB	0.027394786	0.027394786	0.027394786	150	100	15	2000	Duke Lemur Center CC BY-NC
DLC 2921m	*Nycticebus pygmaeus*	Skull Zipped TIFF Stack	https://doi.org/10.17602/M2/M14961	3.32 GB	0.03434173	0.03434173	0.03434173	150	100	15	2000	Duke Lemur Center CC BY-NC
DLC 2921m	*Nycticebus pygmaeus*	Full Body Zipped TIFF Stack	https://doi.org/10.17602/M2/M16598	8.03 GB	0.052322794	0.052322794	0.052322794	150	100	15	2000	Duke Lemur Center CC BY-NC
DLC 2926m	*Nycticebus pygmaeus*	Hand Zipped TIFF Stack	https://doi.org/10.17602/M2/M14962	2.29 GB	0.016929612	0.016929612	0.016929612	155	120	18.6	2000	Duke Lemur Center CC BY-NC
DLC 2926m	*Nycticebus pygmaeus*	Foot Zipped TIFF Stack	https://doi.org/10.17602/M2/M14963	2.78 GB	0.021844195	0.021844195	0.021844195	155	120	18.6	2000	Duke Lemur Center CC BY-NC
DLC 2926m	*Nycticebus pygmaeus*	Skull Zipped TIFF Stack	https://doi.org/10.17602/M2/M14964	4.34 GB	0.036006387	0.036006387	0.036006387	155	120	18.6	2000	Duke Lemur Center CC BY-NC
DLC 2926m	*Nycticebus pygmaeus*	Full Body Zipped TIFF Stack	https://doi.org/10.17602/M2/M16905	5.01 GB	0.05111758	0.05111758	0.05111758	155	120	18.6	2000	Duke Lemur Center CC BY-NC
DLC 2928m	*Nycticebus pygmaeus*	Foot Zipped TIFF Stack	https://doi.org/10.17602/M2/M14992	2.98 GB	0.030041894	0.030041894	0.030041894	155	120	12	2000	Duke Lemur Center CC BY-NC
DLC 2928m	*Nycticebus pygmaeus*	Hands Zipped TIFF Stack	https://doi.org/10.17602/M2/M14999	3.49 GB	0.031106615	0.031106615	0.031106615	155	120	18.6	2000	Duke Lemur Center CC BY-NC
DLC 2928m	*Nycticebus pygmaeus*	Full Body Zipped TIFF Stack	https://doi.org/10.17602/M2/M15005	4.45 GB	0.082177505	0.082177505	0.082177505	155	120	18.6	2000	Duke Lemur Center CC BY-NC
DLC 1715f	*Otolemur crassicaudatus*	Feet Zipped TIFF Stack	https://doi.org/10.17602/M2/M40222	1.02 GB	0.046544357	0.046544357	0.046544357	150	180	27	2000	Duke Lemur Center CC BY-NC
DLC 1715f	*Otolemur crassicaudatus*	Forelimb Zipped TIFF Stack	https://doi.org/10.17602/M2/M40225	1.55 GB	0.052211957	0.052211957	0.052211957	145	201	29.15	2000	Duke Lemur Center CC BY-NC
DLC 1715f	*Otolemur crassicaudatus*	Full Body Zipped TIFF Stack	https://doi.org/10.17602/M2/M40227	1.84 GB	0.097160997	0.097160997	0.097160997	140	358	50.12	2000	Duke Lemur Center CC BY-NC
DLC 1715f	*Otolemur crassicaudatus*	Hindlimb Zipped TIFF Stack	https://doi.org/10.17602/M2/M40229	2.58 GB	0.062711104	0.062711104	0.062711104	140	213	29.82	2000	Duke Lemur Center CC BY-NC
DLC 1715f	*Otolemur crassicaudatus*	Hands Zipped TIFF Stack	https://doi.org/10.17602/M2/M40234	822.53 MB	0.048919875	0.048919875	0.048919875	150	180	27	2000	Duke Lemur Center CC BY-NC
DLC 1715f	*Otolemur crassicaudatus*	Skull Zipped TIFF Stack	https://doi.org/10.17602/M2/M40238	1.9 GB	0.048919875	0.048919875	0.048919875	150	180	27	2000	Duke Lemur Center CC BY-NC
DLC NN_Og01	*Otolemur garnetti*	Feet Zipped TIFF Stack	https://doi.org/10.17602/M2/M15701	2.44 GB	0.042969402	0.042969402	0.042969402	145	180	26.1	2000	Duke Lemur Center CC BY-NC
DLC NN_Og01	*Otolemur garnetti*	Full Body Zipped TIFF Stack	https://doi.org/10.17602/M2/M15702	1.98 GB	0.091776691	0.091776691	0.091776691	150	292	43.8	2000	Duke Lemur Center CC BY-NC
DLC NN_Og01	*Otolemur garnetti*	Hand Zipped TIFF Stack	https://doi.org/10.17602/M2/M15784	820.3 MB	0.037036035	0.037036035	0.037036035	110	307	33.77	2000	Duke Lemur Center CC BY-NC
DLC NN_Og01	*Otolemur garnetti*	Hand Zipped TIFF Stack	https://doi.org/10.17602/M2/M15785	1.58 GB	0.037036035	0.037036035	0.037036035	110	307	33.77	2000	Duke Lemur Center CC BY-NC
DLC NN_Og01	*Otolemur garnetti*	Skull Zipped TIFF Stack	https://doi.org/10.17602/M2/M33809	6.16 GB	0.034613498	0.034613498	0.034613498	110	307	33.77	2000	Duke Lemur Center CC BY-NC
DLC NN_Og02	*Otolemur garnetti*	Hand Zipped TIFF Stack	https://doi.org/10.17602/M2/M14754	1.9 GB	0.027914777	0.027914777	0.027914777	185	184	34.04	2000	Duke Lemur Center CC BY-NC
DLC NN_Og02	*Otolemur garnetti*	Skull Zipped TIFF Stack	https://doi.org/10.17602/M2/M14762	5.7 GB	0.027914777	0.027914777	0.027914777	185	184	34.04	2000	Duke Lemur Center CC BY-NC
DLC NN_Og02	*Otolemur garnetti*	Full Body Zipped TIFF Stack	https://doi.org/10.17602/M2/M33650	4.94 GB	0.108948588	0.108948588	0.108948588	185	184	34.04	2000	Duke Lemur Center CC BY-NC
DLC NN_Og02	*Otolemur garnetti*	Foot A Zipped TIFF Stack	https://doi.org/10.17602/M2/M33652	2.32 GB	0.040458083	0.040458083	0.040458083	185	184	34.04	2000	Duke Lemur Center CC BY-NC
DLC NN_Og02	*Otolemur garnetti*	Foot B Zipped TIFF Stack	https://doi.org/10.17602/M2/M33658	1.75 GB	0.040458083	0.040458083	0.040458083	185	184	34.04	2000	Duke Lemur Center CC BY-NC
DLC 917f	*Perodicticus potto*	Full Body microCT Volume File	https://doi.org/10.17602/M2/M42252	3.19 GB	0.094036871	0.094036871	0.094036871	196	116	22.74	2000	Duke Lemur Center CC BY-NC
DLC 917f	*Perodicticus potto*	Hindlimb microCT Volume File	https://doi.org/10.17602/M2/M42253	1.93 GB	0.068452098	0.068452098	0.068452098	193	122	23.55	2000	Duke Lemur Center CC BY-NC
DLC 917f	*Perodicticus potto*	Skull microCT Volume File	https://doi.org/10.17602/M2/M42254	3.35 GB	0.039785247	0.039785247	0.039785247	169	98	16.56	2000	Duke Lemur Center CC BY-NC
DLC 919m	*Perodicticus potto*	Foot Zipped TIFF Stack	https://doi.org/10.17602/M2/M15690	2.82 GB	0.035009049	0.035009049	0.035009049	155	120	18	1800	Duke Lemur Center CC BY-NC
DLC 919m	*Perodicticus potto*	Hand Zipped TIFF Stack	https://doi.org/10.17602/M2/M15691	914.08 MB	0.038304985	0.038304985	0.038304985	155	120	18	1800	Duke Lemur Center CC BY-NC
DLC 919m	*Perodicticus potto*	Foot Zipped TIFF Stack	https://doi.org/10.17602/M2/M15692	2.37 GB	0.035009049	0.035009049	0.035009049	155	120	18	1800	Duke Lemur Center CC BY-NC
DLC 919m	*Perodicticus potto*	Hand Zipped TIFF Stack	https://doi.org/10.17602/M2/M15693	1.41 GB	0.038304985	0.038304985	0.038304985	155	120	18	1800	Duke Lemur Center CC BY-NC
DLC 919m	*Perodicticus potto*	Full Body Zipped TIFF Stack	https://doi.org/10.17602/M2/M15698	6.86 GB	0.088456817	0.088456817	0.088456817	155	120	18.6	2000	Duke Lemur Center CC BY-NC
DLC 919m	*Perodicticus potto*	Skull Zipped TIFF Stack	https://doi.org/10.17602/M2/M16253	3.4 GB	0.043280069	0.043280069	0.043280069	155	120	18.6	2000	Duke Lemur Center CC BY-NC
DLC NN_Pp01	*Perodicticus potto*	Foot Zipped TIFF Stack	https://doi.org/10.17602/M2/M15768	1.98 GB	0.03499129	0.03499129	0.03499129	155	120	18.6	2000	Duke Lemur Center CC BY-NC
DLC NN_Pp01	*Perodicticus potto*	Hands Zipped TIFF Stack	https://doi.org/10.17602/M2/M15770	2.8 GB	0.036622722	0.036622722	0.036622722	155	120	18.6	2000	Duke Lemur Center CC BY-NC
DLC NN_Pp01	*Perodicticus potto*	Skull Zipped TIFF Stack	https://doi.org/10.17602/M2/M15771	4.24 GB	0.036622722	0.036622722	0.036622722	155	120	18.6	2000	Duke Lemur Center CC BY-NC
DLC NN_Pp01	*Perodicticus potto*	Full Body Zipped TIFF Stack	https://doi.org/10.17602/M2/M32042	7.64 GB	0.07721854	0.07721854	0.07721854	155	120	18.6	2000	Duke Lemur Center CC BY-NC

### MicroCT scanning

A Nikon XT H 225 ST μCT machine at Duke University’s Shared Materials Instrumentation Facility (SMIF) was used for all scans. This machine has a Perkins Elmer AN1620 X-ray detector panel, which provides a 2000 x 2000 pixel field and a 7.5 frames per second readout. All scans were performed using a Nikon 225kV reflection target with a tungsten anode, which has a focal spot size ranging from 3 to 225 μm depending on the power. No filters were used. The voltage for these scans ranged between 75–203 kV and current settings ranged between 59–593 μA. These settings were largely dependent on the density of the specimen (higher density requires higher X-ray power to penetrate the specimen) and proximity of the detector panel to the reflection target (a smaller distance necessitates lower X-ray power to avoid oversaturation).

In order to ensure that each specimen could be used in subsequent research projects, we did not want scanning events to further degrade the specimen through thawing and refreezing. Therefore, we placed non-iodine stained specimens on dry ice in a Styrofoam cooler, stabilized the specimens with radiotransparent foam to prevent specimen movement during the scan, and conducted the scan with the specimens in the cooler. Iodine stained specimens were scanned in various containers and stabilized with radiotransparent foam but were not placed on dry ice. Variation in the resolution of the scans (voxel size) in Table 2 reflects the maximum level of magnification that could be achieved for an anatomical region given the dimensions of each specimen and the scanning container.

**Fig 2:**
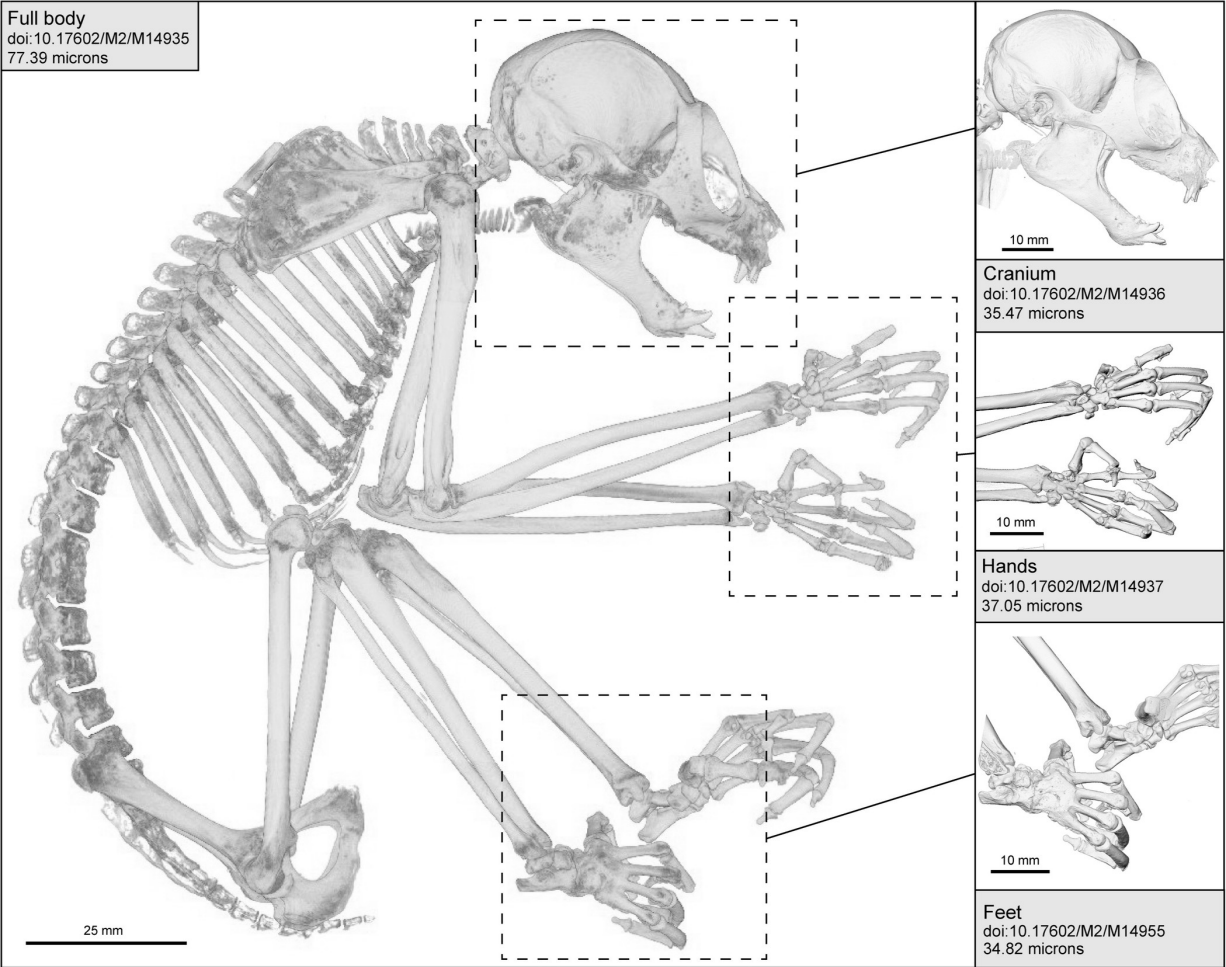
Volume rendering of *Nycticebus coucang* (DLC 2901f) showing scanning protocol for strepsirrhine cadavers. Boxes outline regions of increased anatomical complexity that were scanned at higher resolutions separately. To reduce noise, the threshold for grey values is lower than optimal threshold, rendering less dense bone transparent.

The protocol for this project prioritized comprehensiveness and detail. To that end, most specimens were scanned four to eight times and are represented by four to five volumes: an overview of the full body and separate higher resolution volumes of the skull, hands, and feet (Fig 2). Full body overviews are usually composites created by stitching together TIFF stacks from multiple scanning events (detailed further below).

### Iodine staining

**Fig 3:**
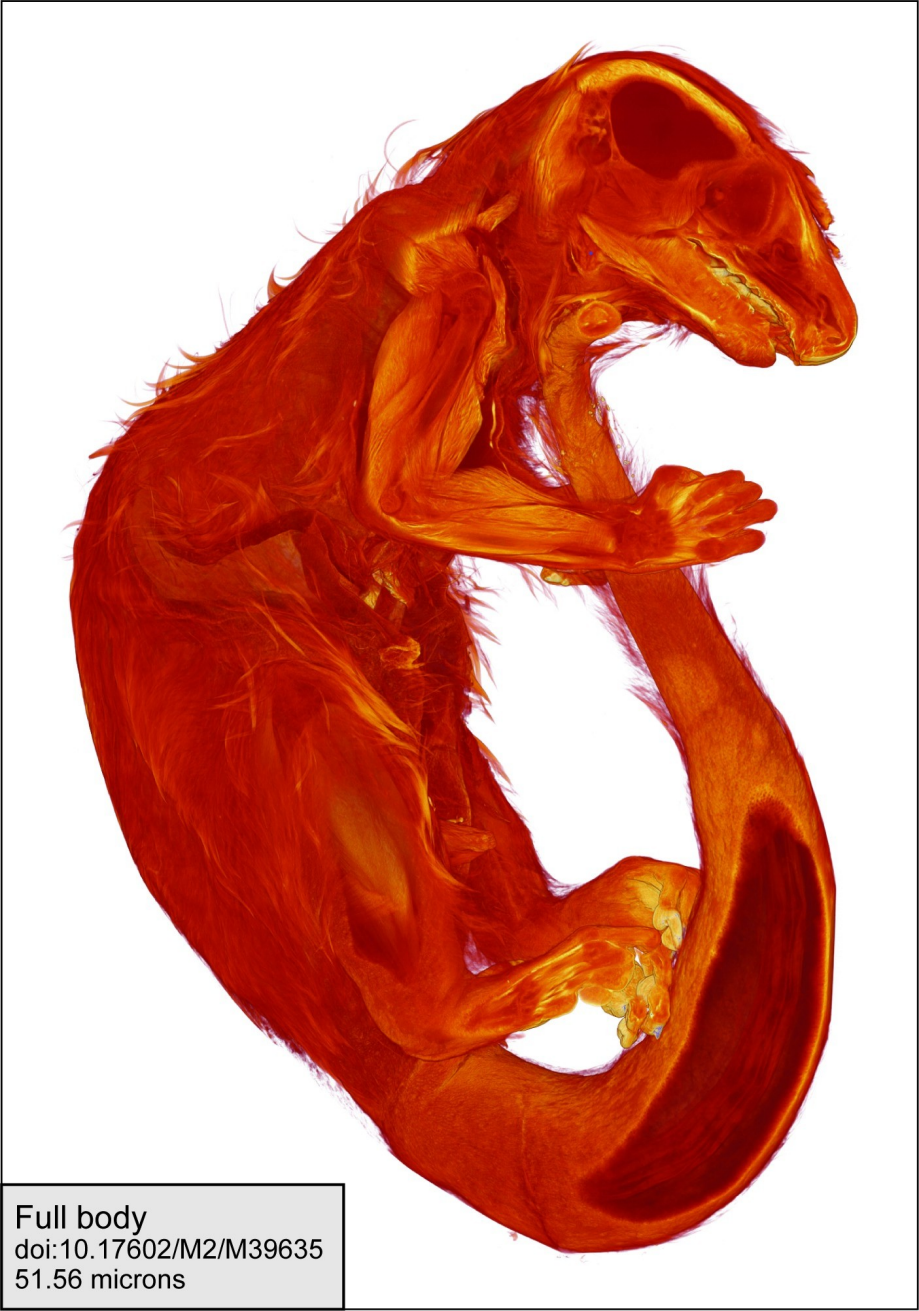
Volume rendering of iodine-stained *Cheirogaleus medius* (DLC 1657m) using Avizo software. A clipping plane in the software digitally slices through the fur and skin to show stained tissues underneath.

The soft tissue anatomy of two lemur and five loris specimens (Table 1) was visualized in the CT-scans using iodine as a contrasting agent [31]. Fig 3 provides an example image from an individual scanned after the staining process. These specimens were thawed and fixed in 10% formalin (Carolina Biological) to prevent deterioration during staining. Specimens were stained in a 7% solution of Lugol’s Iodine (Carolina Biological) for six weeks prior to scanning to allow iodine to penetrate the tissues. Given the large volume of solution required to stain seven cadavers, Lugol’s solution was selected as the contrast-enhancing agent (rather than iosmium-tetroxide [32–34] or phospho-molybdic acid [35]) due to its low toxicity, ease of access, ability to differentially stain types of soft tissue, and cost effectiveness. Following scanning, iodine was removed from the specimens by soaking them in a series of water baths over several weeks to leach the iodine [31] from the specimen’s tissues.

### Post-processing and stitching

X-ray projections were reconstructed as 3D volumes using Nikon XTEK CT Pro 3D version XT 5.3.2, proprietary software purchased with the μCT machine and available to all μCT users at SMIF. Volumes were saved as 16-bit Tagged Image File Format (.TIFF) stacks. High resolutions scans were compressed with 7-Zip software and uploaded to MorphoSource.

**Fig 4:**
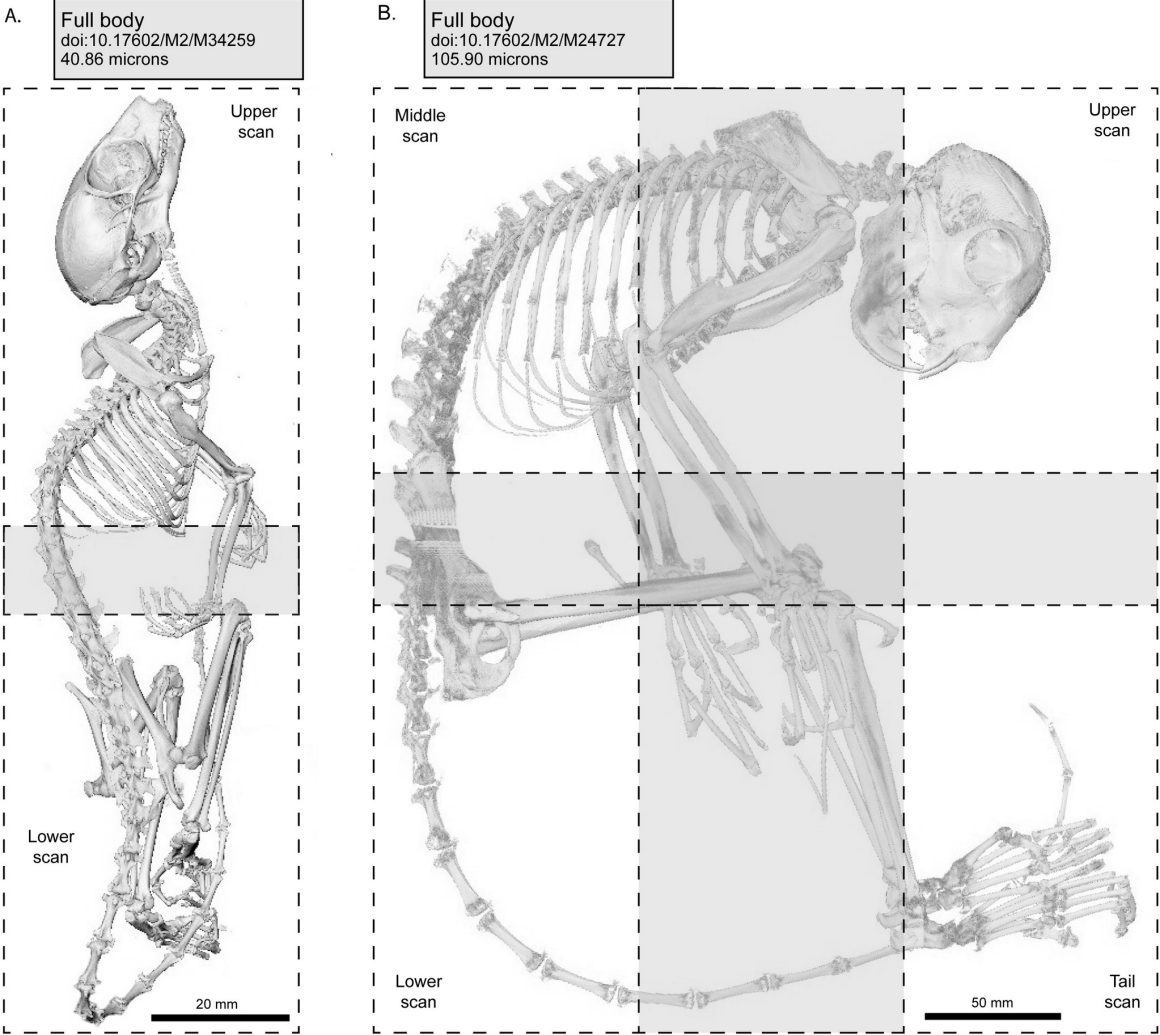
Examples of stitched composite scans. A) composite scan of *Microcebus murinus* (DLC 893m) stitched along a single vertical axis; B) composite scan of *Daubentonia madagascariensis* (DLC 6604m) stitched along two vertical axes. Boxes indicate separate microCT scans, grey areas boxes indicate areas of scan overlap.

If the size of the specimen prevented the full body overview from being done in a single scan, the overview was created by conducting a series of overlapping scans with a shared centre of rotation. This process was easy to accomplish when the specimen was in an extended posture and each scan was oriented along the vertical y-axis (Fig 4A), provided the specimen was not larger than the vertical travel distance permitted by the scanner’s dimensions. However, larger specimens were often flexed into C-shapes that exceeded the dimensions of the detector, even at the lowest magnification. In these cases, scans were conducted as a series of overlapping “panels” with two separate vertical axes (Fig 4B).

To generate full body composites, overlapping scans were stitched together in ImageJ [36] using the 3D Stitching plug-in [37]. As composites of two or more scanning events, the full body overviews are very large volumes. When composite overviews were extremely large (rendering subsequent processing too computationally demanding), we chose to upload the overlapping scans separately. If there are elements partially represented across two scans, these scans can be stitched together by researchers after download.

### MicroCT error study

As discussed by Copes et al. [28], μCT scanners in academic instrumentation facilities accommodate a wide range of users with varying demands for scanning parameters (i.e., detector and stage settings, target type, beam settings). The flexibility required of μCT scanners in academic contexts stands in contrast to industrial or metrology-specific machines, which can be calibrated to maintain a degree of minimum error within a particular set of scan parameters. For scanners in academic instrumentation facilities, accuracy is determined by the initial installation settings and subsequent maintenance, with the assumption that measurement error is around 1%.

Here we expand the calibration study of Copes et al. [28] for SMIF’s Nikon XT H 225 ST μCT machine. To determine the accuracy of the scanner, three different standard spheres of known diameters (3.175mm, 6.35mm, and 12.7mm; machined with a +/- 1.0 μm tolerance) were scanned at a range of voxel resolutions. The 3.175mm and 6.35mm spheres was scanned at 5, 6, 7, 8, 9, 10, 15, and 20 μm per voxel with the detector fixed at its farthest position in the chamber. The 12.7mm sphere was scanned at 10, 20, 30, 40, 50, 60, 70, 80, 90, and 100 μm per voxel with the detector in the same position. Each scan was collected at 175kV, 86μA (15W), 354ms, 2000 projections, 1 frame per projection, and without a filter. Nikon’s proprietary CT Pro 3D and CT Agent software reconstructed the projection data into a volume data file, which was then opened in VG Studio Max 2.2.

In VG Studio, an automatic surface determination was applied to the spheres, >20 fit points were placed on the surface, and an idealized sphere was fit to these points. Diameters produced from this measurement were recorded and compared to the reported diameter of the spheres. The relative percentage error (RE%) was calculated as the difference between the measured (MD) and reported diameters (RD), divided by the measured diameter (RE% = (MD-RD)/MD*100). Given that we are evaluating the same μCT machine, we expect relative % error to be similar to the <0.2% reported by Copes et al. [28].

## Results

### MicroCT error study

**Fig 5:**
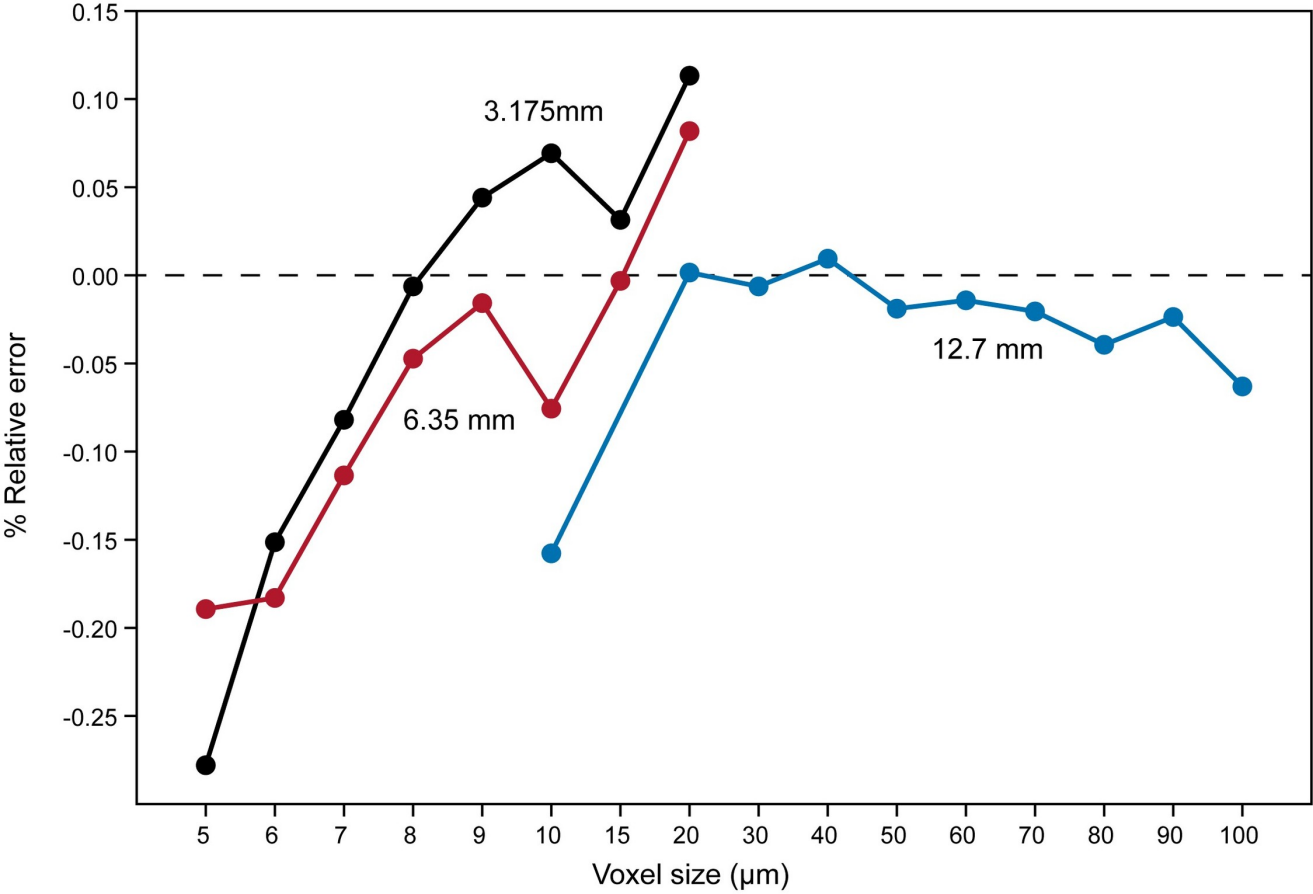
Relative error at different voxel resolutions for three calibration spheres. Spheres of known diameters of 3.175 mm, 6.35 mm, and 12.7 mm and were tested in the Nikon XT H 225 ST μCT scanner at Duke University’s Shared Materials Instrumentation Facility.

Table 3 reports error values for all three standard spheres, and error is plotted against voxel resolution in Fig 5. For each calibration sphere, we found less than 0.3% error at all resolution levels, with most scans demonstrating error levels below 0.1%.

**Table 3:** Relative error at different voxel resolutions for three calibration spheres in the Nikon X-Tek XHT 225 ST scanner at Duke University’s Shared Materials and Instrumentation Facility.

Sphere diameter (mm)	Voxel resolution (μm)	Measured diameter (mm)	Relative error (%)
3.175	5	3.1662	-0.278
	6	3.1702	-0.151
	7	3.1724	-0.082
	8	3.1748	-0.006
	9	3.1764	0.044
	10	3.1772	0.069
	15	3.1760	0.031
	20	3.1786	0.113
6.35	5	6.3380	-0.189
	6	6.3384	-0.183
	7	6.3428	-0.114
	8	6.3470	-0.047
	9	6.3490	-0.016
	10	6.3452	-0.076
	15	6.3498	-0.003
	20	6.3552	0.082
12.7	10	12.6800	-0.158
	20	12.7002	0.002
	30	12.6992	-0.006
	40	12.7012	0.009
	50	12.6976	-0.019
	60	12.6982	-0.014
	70	12.6974	-0.020
	80	12.6950	-0.039
	90	12.6970	-0.024
	100	12.6920	-0.063

### Data records and availability

The μCT scans and associated metadata (Table 2) presented in this project are available through MorphoSource (http://www.morphosource.org), a free-to-use online repository designed to accommodate 3D data and its derivatives, including 3D surface renderings and other digital imagery. MorphoSource was created to address increasing demand for deposition and archiving of 3D digital data representing physical objects, and provides the necessary infrastructure to host, share, and manage 3D data for several different user types—from individual researchers to museum curators—allowing data authors and institutions to benefit from subsequent data use by third parties. Network storage is currently provided by Duke University’s data infrastructure with servers housed in multiple locations. Morphosource is currently supported by Duke University and the National Science Foundation.

The data files of the current project are available open access. Data files are copyrighted by the DLC and can be downloaded and re-used for non-commercial purposes (Creative Commons copyright license CC-BY NC). At the time of manuscript revision, the project had been viewed more than 58,000 times and more than 1900 data files have been downloaded. The most popular downloads have been TIFF volume stacks of *Daubentonia madagascarensis*, a unique and charismatic species not often found in museum collections. Because scanning efforts of the DLC cadaver collection are ongoing, this project will continue to grow to include new specimens and new species.

## Discussion

### Structure of the digital collection

We chose MorphoSource to host this collection because it offers several utilities for data authors and institutions. First, MorphoSource is structured to function as both an online platform for collaborative research and as a public-facing repository for 3D digital data, with high ease-of-use when transitioning between these two roles. For this project, we began to upload scans in 2016 and, for the next two years, data were shared privately with collaborators at multiple institutions. When the project matured, data were made searchable and downloadable to all MorphoSource users, signalling the project’s transition to a publicly facing data collection. In providing this dual functionality, MorphoSource has utility for data authors during all stages of 3D data collection.

Second, MorphoSource provides data authors and institutions multiple ways to track and summarize data usage, including summary reports of data usage as well as the ability to establish citable digital object identifiers (DOIs) for individual scans and derivative data. These tools provide metrics of the value of the collection and help provide professional benefits for researchers undertaking time-intensive data collection efforts. Table 2 was generated from the summary report of this project.

Third, while data collections can be made publicly available through MorphoSource, the platform allows data authors or institutions the option of managing user access through a data request system. This functionality, along with the ability to clearly delineate copyright restrictions and other terms of use, reduces concerns that data will be used in ways that are inconsistent with institutional goals.

Data files on MorphoSource are organized within a three-level hierarchy: specimens > media groups > media files. A fourth organizational unit, the MorphoSource project, exists at the same hierarchical level as the specimen (i.e., specimens are not contained within projects, although media records of a specimen might be). Projects are a collection of media records determined by the user and serve several purposes, including sharing multiple data files with collaborators easily, developing special collections of high public interest (e.g., K-12 Anthropology Teaching Collection, MorphoSource Project P158) or curating institutional holdings (such as this project).

On MorphoSource, specimens are digital representations of physical specimens stored in museums or other collections. As researchers upload new data, they increase the comprehensive sampling of MorphoSource specimens. When new specimen records are created, they can be linked with metadata provided by the home collection of the specimen through iDigBio (http://www.idigbio.org). In the case of this collection, metadata are maintained directly by project members, including DLC staff, as the DLC does not currently serve collection information to data aggregators or use interoperable metadata protocols.

Media groups are nested within specimens and generally represent discrete scanning or other imaging events. Metadata of the imaging event (including scan parameters, calibrations, and funding sources) are attached to media groups.

Media files are nested within media groups and represent raw data (such as TIFF stacks) and derivative data (such as surface renderings). When media files are made publicly available or “published”, they can be assigned DOIs, which function as permanent and direct links to the data as well as references for data citation in subsequent studies. Currently, the DLC cadaver project contains media files tagged by 1329 digital object identifiers. Further detail on the structural organization and associated metadata of MorphoSource can be found in Boyer et al. [24].

### Computer requirements

By current computer standards, these μCT scans are large files. TIFF stacks can range from 1 to 13 GBs and the stitched full body composites are particularly large. System performance depends primarily on four hardware components: the graphics processing unit (GPU), the central processing unit (CPU), the amount of random-access memory (RAM), and the hard drive. For direct volume renderings or 3D surface visualization, the system should have a high-end graphics card (minimally a DDR5 memory interface with more than 2 GB RAM). Sufficient RAM is critical for 3D visualization and analyses; we follow Copes et al. (2016) in recommending computers have RAM that is at least twice as large as the largest TIFF stack. Image processing speed relies on the CPU, so processors with high clock speeds (greater than 3 gigahertz) are recommended. CPU clock speed is particularly important if volume rendering software is unable to access multiple cores. Finally, for uploading datasets quickly, solid state drives (SSD) provide much faster reading and writing speeds than traditional hard disk drives (HDD).

Other recommendations to improve performance for analysing 3D data are a 64-bit operating system and multiple processing cores (provided software compatibility). Specific 3D visualization programs (listed below) may have their own set of system recommendations, so researchers are encouraged to evaluate the match between their preferred software and available workstation.

### Data manipulation

When downloaded from MorphoSource, these μCT scans will first need to be decompressed using open-source programs such as 7-Zip. After decompression, we recommend that users then open TIFF stacks with 2D image processing software such as Fiji [38] or ImageJ [36], as these programs permit some volume editing but require less memory than industrial volume visualization and analysis software. In Fiji or ImageJ, the user can easily adjust grey values to enhance contrast and crop the original μCT volume to regions of interest. Edited TIFF stacks can be saved as new image sequences that require less memory to open and manipulate. For 3D visualization and analyses, there are several commercially available programs (e.g., Avizo, Amira, Dragonfly, Mimix, Osirix, Spiers, and VG Studio Max) as well as freeware (e.g., Slicer3D).

### Citation of scans

Usage terms are outlined in the MorphoSource user agreement that accompanies each download from the website. Subsequent publications that make use of these scans should include 1) a citation of this study; 2) a list or table of DOIs for each scan used; and 3) a statement of access accompanying the DOI list or in the acknowledgments. This statement should read “The Duke Lemur Center provided access to these data under a reuse but non-commercial creative commons license (CC BY-NC), originally appearing in Yapuncich et al. (2019), the collection and archiving of which was funded by NSF BCS 1540421 and NSF BCS 1552848. The files were downloaded from www.MorphoSource.org, Duke University.” A similar statement is included in the accompanying scan metadata.

Although not required by the usage agreement, we would urge researchers who conduct additional processing of these scans (e.g., generate surface files of elements of interest) to upload derivative files to MorphoSource. While we recognize that uploading derivatives can be a time-intensive process, we feel it is a critical component of data sharing and subsequent research. To facilitate this process, bulk upload options have been developed and are available through MorphoSource. Finally, we encourage researchers to contact the DLC research manager to obtain a DLC publication number when manuscripts using these data have been accepted for publication.
